# De novo reconstruction of a functional in vivo-like equine endometrium using collagen-based tissue engineering

**DOI:** 10.1038/s41598-024-59471-z

**Published:** 2024-04-19

**Authors:** Sawita Santiviparat, Theerawat Swangchan-Uthai, Tom A. E. Stout, Supranee Buranapraditkun, Piyathip Setthawong, Teeanutree Taephatthanasagon, Watchareewan Rodprasert, Chenphop Sawangmake, Theerawat Tharasanit

**Affiliations:** 1 Department of Obstetrics, Gynecology and Reproduction, Faculty of Veterinary Science, Chulalongkorn University, Bangkok, Thailand; 2 CU-Animal Fertility Research Unit, Chulalongkorn University, Bangkok, Thailand; 3 Veterinary Clinical Stem Cells and Bioengineering Research Unit, Chulalongkorn University, Bangkok, Thailand; 4 Department of Clinical Sciences, Utrecht University, Utrecht, The Netherlands; 5 Division of Allergy and Clinical Immunology, Department of Medicine, Faculty of Medicine, King Chulalongkorn Memorial Hospital, Chulalongkorn University, Thai Red Cross Society, Bangkok, 10330 Thailand; 6 Faculty of Medicine, Center of Excellence in Vaccine Research and Development (Chula Vaccine Research Center-Chula VRC), Chulalongkorn University, Bangkok, 10330 Thailand; 7 Thai Pediatric Gastroenterology, Hepatology and Immunology (TPGHAI) Research Unit, Faculty of Medicine, King Chulalongkorn Memorial Hospital, Chulalongkorn University, The Thai Red Cross Society, Bangkok, 10330 Thailand; 8 Department of Physiology, Faculty of Veterinary Medicine, Kasetsart University, Bangkok, Thailand; 9 Veterinary Pharmacology and Stem Cell Research Laboratory, Faculty of Veterinary Science, Veterinary Stem Cell and Bioengineering Innovation Center (VSCBIC), Chulalongkorn University, Bangkok, Thailand; 10 Faculty of Veterinary Science, Veterinary Systems Pharmacology Center (VSPC), Chulalongkorn University, Bangkok, Thailand; 11 Department of Pharmacology, Faculty of Veterinary Science, Chulalongkorn University, Bangkok, Thailand; 12 Faculty of Dentistry, Center of Excellence in Regenerative Dentistry, Chulalongkorn University, Bangkok, Thailand

**Keywords:** Equine, Endometrium, ROCK inhibitor, Three-dimensional culture, Biotechnology, Cell biology, Developmental biology, Molecular biology, Stem cells

## Abstract

To better understand molecular aspects of equine endometrial function, there is a need for advanced in vitro culture systems that more closely imitate the intricate 3-dimensional (3D) in vivo endometrial structure than current techniques. However, development of a 3D in vitro model of this complex tissue is challenging. This study aimed to develop an in vitro 3D endometrial tissue (3D-ET) with an epithelial cell phenotype optimized by treatment with a Rho-associated protein kinase (ROCK) inhibitor. Equine endometrial epithelial (eECs) and mesenchymal stromal (eMSCs) cells were isolated separately, and eECs cultured in various concentrations of Rock inhibitor (0, 5, 10 µmol) in epithelial medium (EC-medium) containing 10% knock-out serum replacement (KSR). The optimal concentration of Rock inhibitor for enhancing eEC proliferation and viability was 10 µM. However, 10 µM Rock inhibitor in the 10% KSR EC-medium was able to maintain mucin1 (*Muc1*) gene expression for only a short period. In contrast, fetal bovine serum (FBS) was able to maintain *Muc1* gene expression for longer culture durations. An in vitro 3D-ET was successfully constructed using a collagen-based scaffold to support the eECs and eMSCs. The 3D-ET closely mimicked in vivo endometrium by displaying gland-like eEC-derived structures positive for the endometrial gland marker, Fork headbox A2 (FOXA2), and by mimicking the 3D morphology of the stromal compartment. In addition, the 3D-ET expressed the secretory protein MUC1 on its glandular epithelial surface and responded to LPS challenge by upregulating the expression of the interleukin-6 (*IL6*) and prostaglandin F synthase (*PGFS*) genes (P < 0.01), along with an increase in their secretory products, IL-6 (P < 0.01) and prostaglandin F2alpha (PGF2α) (P < 0.001) respectively. In the future, this culture system can be used to study both normal physiology and pathological processes of the equine endometrium.

## Introduction

Effective management of mare fertility is a cornerstone of economic sustainability within the horse breeding industry^1^. Moreover, the endometrium plays a pivotal role in influencing fertility by orchestrating various critical reproductive functions such as sperm transport, resistance to inflammation, and support of conceptus development^2,3^. Endometrium has a complex 3D structure, comprising hormone-responsive epithelial and stromal cells embedded in a dynamic extracellular matrix (ECM)^4–6^. Different hormonal cues to which the uterus is exposed during the estrous cycle, and alterations as a result of pathological conditions, lead to changes in endometrial architecture that eventually affect endometrial function^7^. Nevertheless, our understanding of the control of the intricate changes in the equine endometrium required to enable sperm transport, clear breeding-induced inflammation and allow maternal recognition of pregnancy and implantation remains unclear.

In vitro culture systems have been shown to provide a valuable framework for investigating equine endometrial function. However, existing methods have notable limitations. For example, two-dimensional (2D) culture systems poorly represent the in vivo structure and function, compared with three-dimensional (3D) culture systems^8^. Although tissue explants preserve the 3D structure, long-term culture leads to cellular degeneration^9^. Furthermore, it is noteworthy that the previous 3D approach omitted the incorporation of an extracellular matrix (ECM), in which cells were cultured on either side of a semipermeable membrane^10^. Additionally, while the 3D equine endometrial organoid employed an ECM (Matrigel ®)^11^, the emphasis on cellular residency, cell–cell communication, cell-ECM interactions and endometrial function remains inadequately addressed. This is largely due to the absence of a model accurately representing the complexity of in vivo endometrial tissue. Therefore, the development of an equine in vitro 3D-ET using tissue engineering technology, and incorporating both endometrial cells and the ECM, will more closely represent the in vivo tissue. This innovative technology could serve as a valuable model for investigating endometrial function in both the horse and other species, such as man^12^.

The quality and differentiation capacity of primary eECs are also critical factors in the development of an effective in vitro 3D-ET. A selective Rho-associated coiled-coil kinase (ROCK) inhibitor (Y-27632) has been shown to play a pivotal role in supporting various cellular regulatory processes in diverse epithelial cell types, including the promotion of proliferation, migration, differentiation, and the suppression of apoptosis^13–18^. In the field of endometrial research**,** Y-27632 is commonly supplemented into culture medium for enhancing murine and human organoid formation and dissociation^19–21^. Nevertheless, the efficacy of Y-27632 differs across cell types and is dose dependent^22^. In horses, information on the effect of Y-27632 on eEC proliferative activity and function is limited. Therefore, investigating the effects and optimizing the concentration required to enhance the quality of equine eECs could help to improve the overall quality of a putative 3D in vitro endometrial model. This study aimed to investigate the optimal culture conditions and ROCK inhibitor concentration for equine eECs to be incorporated into a 3D-ET. The resulting in vitro 3D-ET was also examined in terms of structure and function, using a lipopolysaccharide (LPS) induced inflammation model.

## Methods

**Figure 1:**
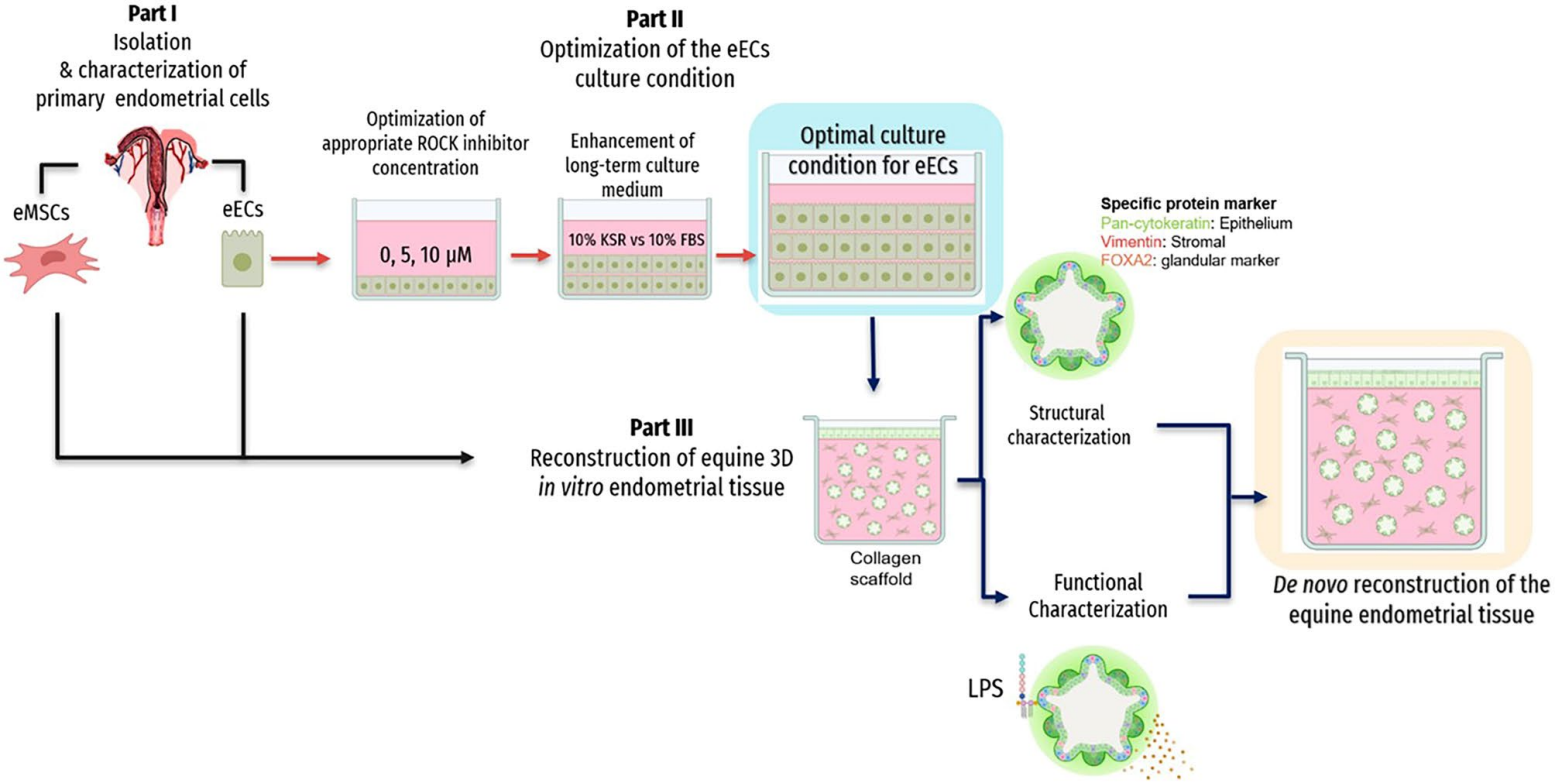
A schematic diagram of the three different parts of this study.

The animal care and use protocol was approved and performed according to the CU-ACUP of Chulalongkorn University (protocol 2131028) which complied with the ARRIVE guidelines. (The full form of CU-ACUP was in the Supplementary Document.) The biosafety procedures followed the guidelines of the Institutional Biosafety Committee of the Faculty of Veterinary Science, Chulalongkorn University (No. IBC1631022). The schematic diagram of materials and methods in this study was illustrated in Fig. 1.

### Isolation and characterization of equine eECs and eMSCs

#### Animals and sample collection

Eight healthy cyclical mares (1.5–7 years old) with normal estrus cycles were selected. The estrus cycles were consecutively monitored during breeding season by transrectal palpation and ultrasonography of the ovaries and uterus in order to detect the ovulation day (Day 0)^23^. Subsequently, the uterine biopsy was performed on days 2–7 post ovulation. The mares underwent anesthesia using 0.01 mg/kg detomidine. Pre-operative analgesia was administered intravenously with butorphanol tartrate at a dosage of 0.1 mg/kg. Additionally, localized epidural anesthesia was provided using 2% lidocaine. The biopsy samples were collected using EQUIVET uterine biopsy forceps (Cat. No 141965, KRUUSE), and transported at 26–28 °C to the laboratory within 6 h in a 0.9% saline solution supplemented with antibiotics (100 IU/ml penicillin and 100 µg/ml streptomycin). Post-operative care included four days of intravenous Flunixin meglumine (1.1 mg/kg) for pain management. The endometrial tissue biopsies were evaluated and categorized according to Kenney^24^ as part of the screening process for reproductive health before isolating the eECs and eMSCs. Tissues classified as grade I were chosen for further analysis.

#### Isolation of the eECs and eMSCs

The endometrial tissue was first cut into 3 × 3 mm pieces and cultured as explants in a petri dish using specific culture media: eMSCs medium and either semi-defined or defined epithelial medium. The eMSCs medium included 88% low glucose Dulbecco’s Modified Eagle Medium (DMEM, 31600-034, Gibco®), 10% (v/v) fetal bovine serum (FBS, 10270-106, Gibco®), 1% (v/v) l-glutamine (25030-081, Gibco®), and 1% (v/v) antibiotic/antimycotic solution 100× (15240-062, Gibco®). The epithelial medium, known as FBS-EC medium, featured MSC medium supplemented with 0.01 µg/ml epidermal growth factor (EGF) and 2.436 mM hydrocortisone. Alternatively, a defined culture medium, KSR-EC medium, was used that was similar to FBS-EC except that the FBS was replaced by 10% (v/v) knockout serum replacement (KSR). The explanted tissues and cells were incubated at 37 °C in a humidified atmosphere of 5% CO_2_-in-air until the expanded cells reached 80–90% confluence. Subsequently, the cell outgrowths were isolated by time-dependent trypsinization. Stromal cells underwent digestion for 2 min, while epithelial cells were digested for 5 min by exposure to 0.25% trypsin EDTA (25200-072, Gibco) at 37 °C. Following trypsinization, the two cell types were cultured separately to purify the population in their respective specific culture media. Eventually, the cells were sub-cultured and then trypsinized and cryopreserved at − 80 °C in the cryopreserved medium containing 10% (v/v) DMSO and 90% (v/v) fetal bovine serum. The morphology of eECs and eMSCs was examined under a phase contrast microscope (CK X41 Olympus, Japan).

#### Characterization of eECs and eMSCs

The eECs and eMSCs were characterized using a modification of the approach described by Rink et al.^25^. Immunofluorescence was used to detect proteins specific for eECs (Pan-cytokeratin) or for eMSCs (Vimentin). Flow cytometry was then used to assess eEC purity after staining. The eECs were gated according to side scatter to assess cellular complexity or granularity, and forward scatter to indicate cell size. Subsequently, the same population from both scatter sides within each cell line was selected for analysis. The samples were analyzed utilizing the BDFACSCalibur flow cytometer (BD, USA) with the BD CellQuest™ Pro software. Conventional polymerase chain reaction (conventional PCR) was also used to detect the expression of genes specific to eECs (*Muc1*) and eMSCs (*CD29*, *CD44*, *CD90*). For eMSCs, adipogenic and osteogenic differentiation was induced following the criteria outlined for MSC properties described by the International Society for Cellular Therapy^26^. Tissue-specific gene expression markers (*LPL* for adipogenic and *COL1A1* for osteogenic) were assessed using conventional PCR. Alizarin red staining was used to detect extracellular calcium deposits during osteogenic differentiation, and alkaline phosphatase (ALP) activity was measured using an alkaline phosphatase test kit. Oil-red O staining was used to visualize lipid droplets in cells differentiated towards an adipogenic phenotype. This characterization approach confirmed the distinct identities of the eECs and eMSCs. Additional details of this process are illustrated in the Supplementary Material.

##### Primer design, RNA extraction and conventional PCR

Primer sequences for target genes were designed using Primer3 Web-based software in the National Center for Biotechnology Information (NCBI; http://www.ncbi.nlm.nih.gov/) with reference to Rink et al.^25^, as illustrated in Table 1; expression of a housekeeping gene (*GAPDH*), an epithelial marker (*Muc1*), and MSC markers (*CD29*, *CD44*, *CD90*), osteogenic markers and adipogenic markers were all examined. The RNA extraction and protocol for conventional PCR were modified from Setthawong et al.^27^, and described in the Supplementary Material.

**Table 1:** Primer pairs for conventional and real-time PCR of genes expressed by equine endometrial epithelial cells (eECs) and mesenchymal stromal cells (eMSCs) (*see Rink et al.^25^.

Primer name	Sequence	Accession number	Annealing temperature (°C)	Amplicon size (bp)
*BAX*	F: TTT GCT TCA GGG TTT CAT CC R: ATC CTC TGC AGC TCC ATG TT	XM_001489207.1	53	197
*BCL2*	F: GAG ACC CCC AGT GCC ATC AA R: GGG ATG TCA GGT CGC TGA AT	XM_001499714.1	58	233
*COL1A1*	F: TGC GAA GAC ACC AAG AAC TG R: GAC TCC TGT GGT TTG GTC GT	XM_023652710.1	56	108
CD29*	F: GGC TAA CAG GGA GTT TCA GAT R: ACA TCT ATT TTC ATC TGC TTG GC	NM_001301217.1	53	100
CD44*	F: CCC ACG GAT CTG AAA CAA GTG R: TTC TGG AAT TTG AGG TCT CCG TAT	XM_023653788.1	58	95
CD90*	F: TGC GAA CTC CGC CTC TCT R: GCT TAT GCC CTC GCA CTT G	XM_023644995.1	53	93
*Foxa2*	F: GTG ATT GCT GGT CGT TTG TTG R: TGT TCA TGC CGT TCA TCC C	XM_023626003.1	55	186
*IL6*	F: CAA TCT GGG TTC AAT CAG GA R: GAA GGA TGA GGT GAG TTG TT	NM_001082496.2	53	277
*LIMK1*	F: AGA GGA AGG AAG CGA GTT GC R: CCA TGA CCA GCC CCT TAG TG	XM_023655469.1	56	245
*LIMK2*	F: GTT TAT CTG GTG TGG GAA GAA G R: CTG GGT GGT ACT TGA ACT CC	XM_001497153.5	56	239
Muc1*	F: CTA TCT CGT TGC CCT GGC TG R: GTA GGC ATC ACG GGT TGG AA	XM_023641050.1	58	82
*RHOA*	F: CGA TTC TCT CGT TGG TCC R: TCC TTG CTA AAG ACG ATG AG	XM_023619803.1	50	280
*ROCK1*	F: ACT GGA AAA CTG ATT CGT CT R: TCA GCC ATG AGA AAA CAC C	NM_001163985.2	50	192
*ROCK2*	F: AGA GAT TTC ACC AGC ACA TT R: CCA GGT ACA CAA ATG AGT GA	XM_023619528.1	51	300
*PGFS*	F: TGG GTT CCG CCA TAT TGA TT R: CAA CTC GGG TCG AAG GAA AG	NM_001081895.2	55	151
*LPL*	F: ACG TCT TAC ACA CAT TCA CC R: TTG GAA AGT GCC TCC ATT AG	AB278557	53	101
GAPDH*	F: GAA GAT GTG GCG CGA TGG CC R: ACT GAC ACG TTA GGG GTG GGG AC	NM_001163856.1	65	147

### Optimization of culture conditions for eECs using the ROCK inhibitor, Y-27632

The effect of the ROCK inhibitor, Y-27632, on eECs derived from the defined culture medium (KSR-EC medium; n = 3 cell lines) was analyzed. The cells were first examined by conventional PCR in order to validate the presence of the Rho/ROCK pathway. The eECs cells were then cultured in a medium supplemented with different concentrations of Y-27632 (0, 5, and 10 µM). The Effect of the ROCK inhibitor on eECs function was determined via the following experiments.

#### Detection of Rho/ROCK pathway in eECs

##### Conventional-PCR

Expression of genes associated with the Rho/ROCK downstream pathway (*RHOA*, *ROCK1*, *ROCK2*, *LIMK1*, and *LIMK2*) was detected using conventional PCR. Primer sequences were derived from NCBI and listed in Table 1. Samples were amplified using 100 ng/ml of cDNA. conventional PCR was performed following a previously established protocol.

#### Proliferation assay

##### Doubling time

eECs (15,000 cells/well) were cultured in triplicate in a 24-well plate with varying concentrations of Y-27632 at 37 °C in a humidified atmosphere of 5% CO_2_-in-air. At 24, 48, and 72 h, cells were detached from the plastic by trypsinization with 0.25% trypsin–EDTA at 37 °C for 5 min, and cell number was determined by staining with trypan blue followed by counting with a hemocytometer. Doubling time was calculated using V. Roth MD's Doubling Time Calculator (2006).

##### Edu assay staining

The effect of Y-27632 on de novo DNA synthesis was evaluated after 72 h of culture using Click-it Edu (Click-iT™ Edu Alexa Fluor™ 488 Imaging Kit, Lot 1939601 (Invitrogen by Thermo Fisher Scientific, Oregon USA) Cell proliferation assays following the manufacturer’s instructions. Briefly, the Edu labeling was added at 48 h (1 µl/1 ml of culture medium) and incubation was continued for a further 24 h. At the end of the 24 h incubation with label, all samples were collected and permeabilized. The Click-it reaction cocktail was added into the cell pellet (500 µl/cell line) which was incubated for 1 h at room temperature, with light protection. Flow cytometry was used to detect the Edu (labelled with AlexaFluor 488® azide) with the help of a green emission filter (wavelength 519 nm). The proliferation rate was calculated as the proportion of Edu-positive nuclei among the cells.

#### Viability and apoptosis validation

##### FITC annexin V-propidium iodide staining

The eECs were cultured in triplicate (50,000 cells/cm^2^) in a 24-well plate with varying Y-27632 concentrations (0, 5, 10 µM). Apoptosis was induced by exposing the eECs to 200 mM hydrogen peroxide (H_2_O_2_) for 24 h^28^. Subsequently, all samples were collected and stained with the FITC-Annexin V Apoptosis Detection Kit with PI (Lot B337692, BioLegend®) according to the manufacturer’s protocol, and flow cytometry was used to assess the percentage of viable cells.

##### Quantitative PCR (qPCR, real-time PCR)

The expression of genes related to apoptosis (Pro-apoptotic: *BAX*, and Anti-apoptotic: *BCL2*) was assessed in control and treatment eEC populations by real-time PCR. RNA extraction and cDNA conversion were performed as described previously. The primer sequences for *BAX* and *BCL2* are listed in Table 1. *GAPDH* was selected as a reference gene. For optimization of the qPCR, the primer was initially tested by conventional PCR. Standards for the qPCR were prepared from purified PCR products using a QIAquick PCR purification kit (QIAGEN) that was quantified by spectrophotometry and diluted over at least 9 orders of magnitude. The standard curve was achievable with R^2^ > 0.995 and the efficiency of the reaction close to 1.0 which means that 100% cDNA has occurred after each cycle. Real-time PCR was performed following the protocol described by Swangchan-Uthai^29^. All experiments were repeated in duplicate with the KAPA SYBR® FAST qPCR kit, KAPA Biosystems. The relative expression was developed from Livak and Schmittgen^30^, then levels of the *BAX* and *BCL2* genes were normalized to GAPDH. Data of relative gene expression and the ratio of *BAX*: *BCL2* were expressed as means ± SEM and compared by one-way ANOVA.

#### Functional assessment of equine endometrial cells (eECs)

For functional assessment, the eECs were cultured under optimal Y-27632 conditions in the defined KSR-EC medium. As determined by the expression of the specific functional secretory gene *Muc1*, these cells could maintain functional status up to passage 6, with the chosen ROCK inhibitor concentration. Confirmation was based on conventional PCR to examine the expression of the *Muc1* gene.

#### Effect of fetal bovine serum on eECs proliferative activity

Due to limited proliferation of eECs when cultured in KSR-EC medium, the effect of FBS on cell proliferation was examined. The eECs were cultured in 10% FBS-EC medium containing the optimal ROCK inhibitor concentration. The cells were then further sub-cultured (Passage 6). Conventional PCR was used to analyze *Muc1* gene expression.

### Reconstruction of equine in vitro 3D-ET

For this experiment, the 3D-ET was maintained under the optimal long-term culture conditions (FBS-EC medium with 10 µM Y-27632) established in the preceding experiment.

#### Collagen scaffold preparation

The sterilized collagen basement membrane (BM) (Nitta Gelatin Inc., Japan, Lot no. 190709) was neutralized using 200 mM HEPES buffer and adjusted to pH 7–7.5 with 10 mM sterile NaOH. The collagen was gently stirred using a sterilized magnetic stirrer for approximately 5 min and maintained at 4 °C until used.

#### Reconstruction of the 3D-ET

Before reaching confluency, the eECs and eMSCs at passages 3–4 were digested and mixed at a ratio of 1:1 and then centrifuged at 157×*g* at 4 °C for 5 min to generate a cell pellet. After removing the supernatant, the prepared endometrial cells were mixed gently with the collagen (total of 150,000 cells/well) volume (500 µl or 0.965 cm^3^). This mixture was carefully introduced into a 24-well plate, followed by the addition of 200 µl of PBS to cover the collagen within each well. The plate was then placed in a 37 °C incubator in a humidified atmosphere of 5% CO_2_-in-air for 1.5 h to allow the collagen to polymerize. After gel formation, the PBS was removed and 500 µl of 10% FBS-EC medium was added into each well. On day 3 of culture, the supernatant was removed, the collagen was washed with PBS, and a layer of Matrigel™ was overlain onto the collagen as an artificial basement membrane and incubated at 37 °C for 15 min. Subsequently, eECs were seeded onto the Matrigel™, at approximately 1.5 × 10^4^ cells/cm^2^ This culture configuration facilitated cell growth over a 7–14 day period, with medium renewal every 2–3 days. Morphological changes in the reconstructed tissue were monitored daily using a phase contrast microscope (CK X41 Olympus, Japan).

#### 3D-ET characterization

##### Specific gene and protein characterization

*Conventional PCR* Expression of the endometrial gland marker (*Foxa2*) and the secretory marker (*Muc1)* in in vitro 3D-ET were assessed via conventional PCR. The primer sequences are listed in Table 1. The conventional PCR protocol was conducted as described previously.

*Immunofluorescent and confocal microscopy* The whole section of in vitro 3D-ET was fixed by immersion in 4% paraformaldehyde (PF) solution for 30 min. Subsequently, the in vitro 3D-ET was cut with a surgical blade into 3 × 3 mm pieces and placed onto glass slides. To identify the eECs and eMSCs, antibodies against Pan Cytokeratin and Vimentin were used, as described previously. Wheat germ agglutinin was additionally used to identify the cell membrane boundary. The nuclei were stained with 4′,6-diamidino-2-phenylindole (DAPI). For the identification of endometrial gland-like structures, a primary antibody against FOXA2 was used (see Table 2 for details of primary and secondary antibodies). Additionally, the secretory function of the endometrial gland-like structures was assessed by staining for MUC1 (sc-7313, Santa Cruz Biotechnology), with a protocol adapted from Piña et al.^31^. The details are described in the Supplementary Information. The anti-fade reagent was applied to the in vitro tissue blocks before they were covered with a cover glass and mounted using a mounting solution. Visualization of the overall structure was achieved using an immunofluorescent microscope, whereas z-stacks created via a confocal microscope (AX/AXR with NSPARC, Nikon Japan) were used for 3D structure assessment.

**Table 2:** Antibodies used to characterize eECs, eMSCs, and to detect endometrial markers.

Marker	Primary antibody	Secondary antibody
Pan Cytokeratin	Rabbit monoclonal anti-Pan Cytokeratin (ab234297, Abcam) (1:50 dilution)	Monoclonal anti-rabbit IgG FITC (ab6717, Lot:GR3237804-1, abcam) (1:50 dilution)
Vimentin	Mouse monoclonal anti-Vimentin (V5255, Sigma Aldrich) (1:50 dilution)	Anti-mouse IgG TRITC (T5393-1ML, Lot#SLBJ3612V, Sigma) (1:100 dilution)
FOXA2	Mouse monoclonal IgG1 κ HNF-3β Antibody (H-4) (sc-374376, Santa Cruz Biotechnology) (1:50 dilution)	Anti-mouse IgG TRITC (T5393-1ML, Lot#SLBJ3612V, Sigma) (1:100 dilution)
MUC1	Monoclonal IgG1 κ Mucin 1/MUC1 antibody (sc-7313, Santa Cruz Biotechnology) (1:50 dilution)	Anti-mouse IgG TRITC (T5393-1ML, Lot#SLBJ3612V, Sigma) (1:100 dilution)

##### Functional testing of 3D-ET via a lipopolysaccharide (LPS) challenge

For functional testing, the 3D-ETs were divided into triplicates. The control group was cultured in normal 10% FBS EC-medium, whereas the treatment group was cultured in FBS-EC supplemented with *Escherichia coli* O55:B5 bacterial LPS (5 µg/ml, Sigma Chemical Inc.). All 3D-ETs were incubated at 37 °C in 5% CO_2_-in-air for 24 h. After the 24 h incubation, the culture medium and in vitro 3D-ET samples were collected separately and frozen at − 80 °C for subsequent investigations. Enzyme-linked immunosorbent assays (ELISA) were employed for the quantitative analysis of secretory products (IL6 and PGF2α) in the culture media. qPCR was used to determine and quantify gene expression for the inflammatory cytokine *IL6* and for the prostaglandin synthase enzyme, *PGFS,* within the collected in vitro 3D-ET samples (for primers see Table 1).

*ELISA* IL6 concentrations in the medium from the in vitro 3D-ETs was measured in duplicate using the Human Th Cytokine Panel 13-plex assay (Biolegend, USA) and analyzed via flow cytometry (BD FACS Calibur, Becton Dickinson, USA). The full protocol is described in the supplementary materials.

The PGF2α concentration was determined in duplicate using an ELISA Kit (Abnova) in accordance with the manufacturer’s guidelines. The intra-assay coefficient of variation (CV) was 2.82%, and the detection limit was 0.326 pg/ml.

### Statistical analysis

Statistical analysis was performed using SPSS version 29.0.0.0, and all graphs were produced using GraphPad Prism 9.5.1. Continuous data were tested for normality and equivalence of variance and reported as means ± SEM. ANOVA was used to determine differences between treatments in experiments II and III. Independent sample t-tests were conducted to compare relative gene expression for *IL6* and *PGFS*, and secretion of PGF2α in LPS-treated versus non-treated in vitro 3D-ET. Chi-square test of Fisher’s exact test was used to examine the effect of LPS on IL6 secretion.

## Results

### Isolation and characterization of equine eECs and eMSCs

**Figure 2:**
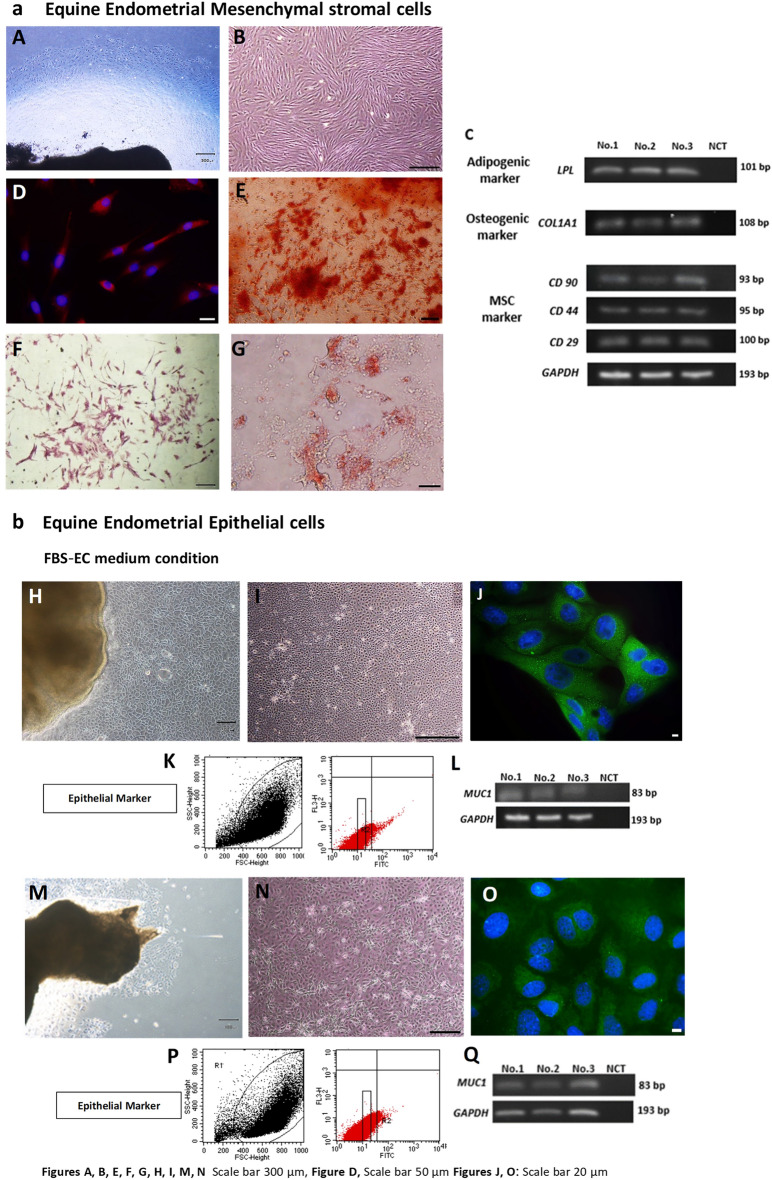
Characterization of eMSCs (**a**) and eECs (**b**). (**A**) The outgrowth of the mix population from the explanted tissue under MSC medium condition. (**B**) The eMSCs had a spindle-shaped morphology, and (**D**) positive Vimentin staining indicated the presence of this intermediate filament protein (red: nuclei in blue). (**C**) MSC-specific markers (*CD29*, *40*, *90*) were demonstrated by gene expression analysis. (**E**) As indicators of osteogenic potential, eMSCs exhibited positive ALP staining, and (**F**) positive alizarin red staining for extracellular calcium deposits under osteogenic culture conditions, confirming differentiation into osteogenic lineages. (**G**) Oil Red O staining emphasized the presence of intracellular lipid droplets, confirming adipogenic differentiation capability of the eMSCs. The characterization of eECs (**b**) revealed (**H**,**K**) The outgrowth of the epithelial cells from the explanted tissue under FBS-EC and KSR-EC medium condition respectively (**I**,**N**) a polygonal cell shape in both EC media tested (FBS-EC and KSR-EC, respectively). (**J**,**O**) Positive Pan Cytokeratin staining of the cytoskeleton was evident in eECs in both media. In FBS-EC media, eEC purity, determined by flow cytometry, was 94.78% (**K**), whereas in KSR media, purity reached 97.70% (**P**). (**L**,**Q**) PCR validated the gene expression for the eEC-specific marker *Muc1* in eECs derived from both EC media. (Uncropped conventional PCR gels were shown in the Supplementary Uncropped Fig. 1A–F for eMSCs and Figure for eECs).

The eMSCs and eECs were isolated from outgrowths from tissue explants, using specific medium conditions. The eMSCs displayed a spindle-shaped morphology, as expected for mesenchymal stem cells, with positive staining for the intermediate filament protein, vimentin. Additionally, these cells exhibited gene expression for specific MSC markers (*CD29*, *CD44*, *CD90*) and demonstrated the ability to differentiate into both osteogenic and adipogenic lineages. In contrast, the eECs obtained from both EC medium conditions (FBS-EC and KSR-EC) exhibited a polygonal cell shape and stained positive for a pan-cytokeratin antibody, indicating epithelial cell characteristics. The flow cytometric analysis indicated that the eECs had a purity of 80–90%. Furthermore, gene expression for a specific marker for eECs (*Muc1*) was evident in eECs derived from both EC medium conditions. Additional details about eECs and eMSCs are shown in Fig. 2.

### Optimization of equine eEC culture conditions

#### The Rho kinase/LIMK kinase downstream cascade pathway is expressed in eECs

**Figure 3:**
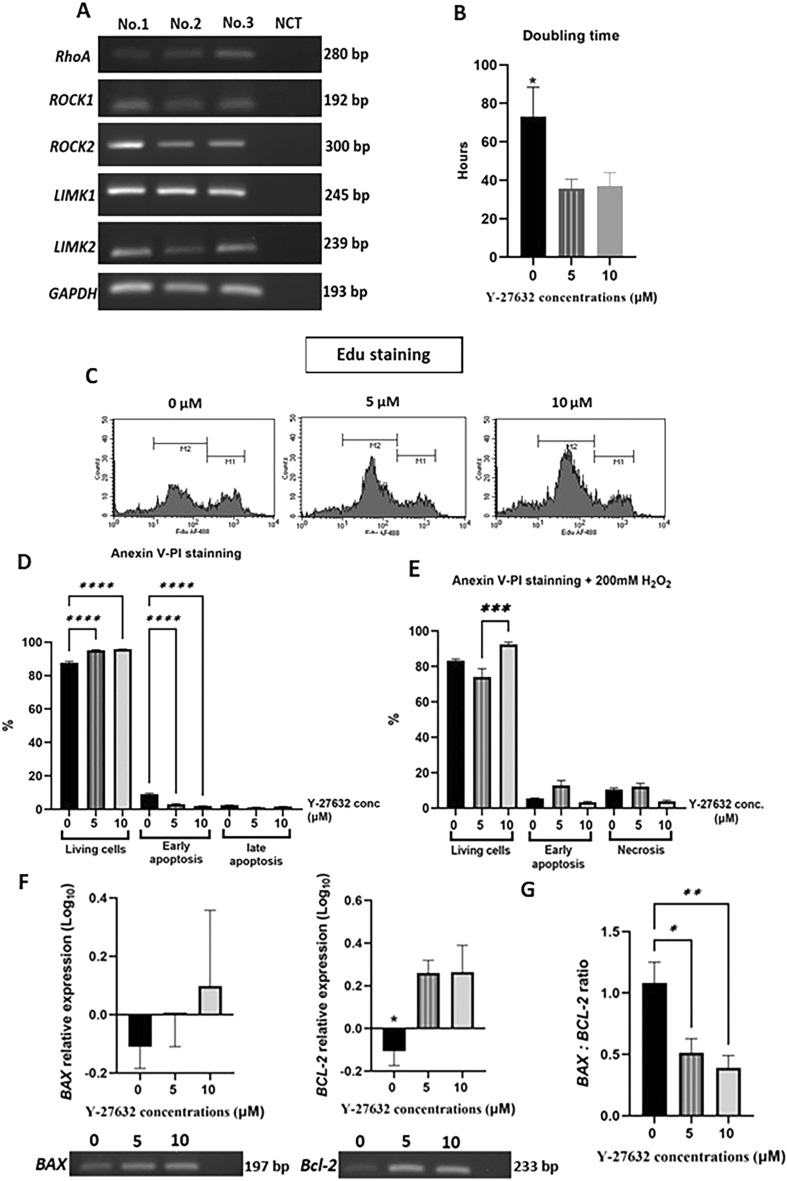
(**A**) Gene expression for Rho kinase/LIM Kinase and downstream cascade in cultured equine endometrial epithelial cells (eECs). (**B**) The doubling time graph indicates more rapid proliferation in the Rho-kinase inhibitor (Y-27632) treated groups. (**C**) Edu labeling highlighted trended toward increasing M2 generation for 10 (3.5–9.1-fold), 5 (2.8–7.1-fold) and 0 (1.7–8.3-fold), (**D**) The Y-27632 treated groups showed enhanced eECs viability and decreased rates of early apoptosis. (**E**) After H_2_O_2_-induced apoptosis, 10 µM Y-27632 treatment resulted in a higher percentage of viable cells and a trend towards a lower incidence of early apoptosis. (**F**) The treated groups exhibited a significant increase in relative log expression of *BCL2* compared to controls, whereas (**G**) the relative log expression for the *BAX* gene did not differ between groups. The *BAX*: *BCL2* ratio was significantly higher in the control group. (Uncropped conventional PCR gels were shown in the Supplementary uncropped PCR Figs. 3, 4).

The expression of downstream genes associated with the ROCK pathway was detected in all eECs cell lines (n = 3) by conventional PCR (Fig. 3). This expression indicated the presence of an active Rho Kinase/LIM Kinase pathway within the eECs.

#### ROCK inhibitor, Y-27632 enhances eEC proliferation

In the treatment groups (5 and 10 µM Y-27632), the doubling times were reduced significantly (42.07 ± 7.66 h and 37.08 ± 6.88 h, respectively) compared with the control group (0 µM; 73.03 ± 15.34 h, P < 0.05). Additionally, Edu labeling indicated a higher percentage of cells with newly synthesized DNA in the 10 µM Y-27632 group (M2), with the control group exhibiting the lowest level of Edu labeling (Fig. 3).

#### ROCK inhibitor, Y-27632, enhances eECs viability and reduces apoptosis

Annexin V-PI staining revealed a significant increase in the percentage of viable cells in the treated eECs groups (5 µM; 95.19 ± 0.39 and 10 µM; 95.83 ± 0.16) compared to the control (0 µM; 87.74 ± 0.77). In addition, there was a marked decrease in the percentage of cells exhibiting early apoptosis in the treated groups (5 µM; 3.16 ± 0.40 and 10 µM; 2.07 ± 0.13) compared to the control (0 µM; 9.02 ± 0.69) (P < 0.0001). After apoptosis induction, the 10 µM group (92.37 ± 1.36) retained a significantly higher percentage of viable cells than the 5 µM Y-27632 group (74.01 ± 4.71, P = 0.0002), although this was not significant than the control (83.27 ± 0.92). The 10 µM Y-27632 group (3.29 ± 0.62) also tended (P = 0.0735) to have a lower incidence of early apoptosis than the 5 µM Y-27632 group (12.86 ± 2.74). However, the percentages of late apoptotic cells did not differ between Y-27632 concentrations.

The Y-27632 treated groups (5 and 10 µM) showed a significant (P < 0.05) increase in the relative log expression of the *BCL2* gene compared to the control group, but there was no significant difference in the relative log expression of *BAX* between treatment groups. However, the *BAX*: *BCL2* ratio was significantly (P < 0.01) lower in the Y-27632 treated groups compared to the control. In this respect, the 10 µM treatment (P = 0.0093) induced a greater decrease in fold difference than the 5 µM treatment (P = 0.0237). These results are summarized in Fig. 3.

Following an assessment of the effects of the ROCK inhibitor on the proliferation and viability of eECs, it was concluded that 10 µM of Y-27632 was the most effective concentration for enhancing the quality of eECs. This concentration was chosen for the culture of eECs in subsequent experiments.

#### Enhancement of long-term culture conditions for eEC cultivation

**Figure 4:**
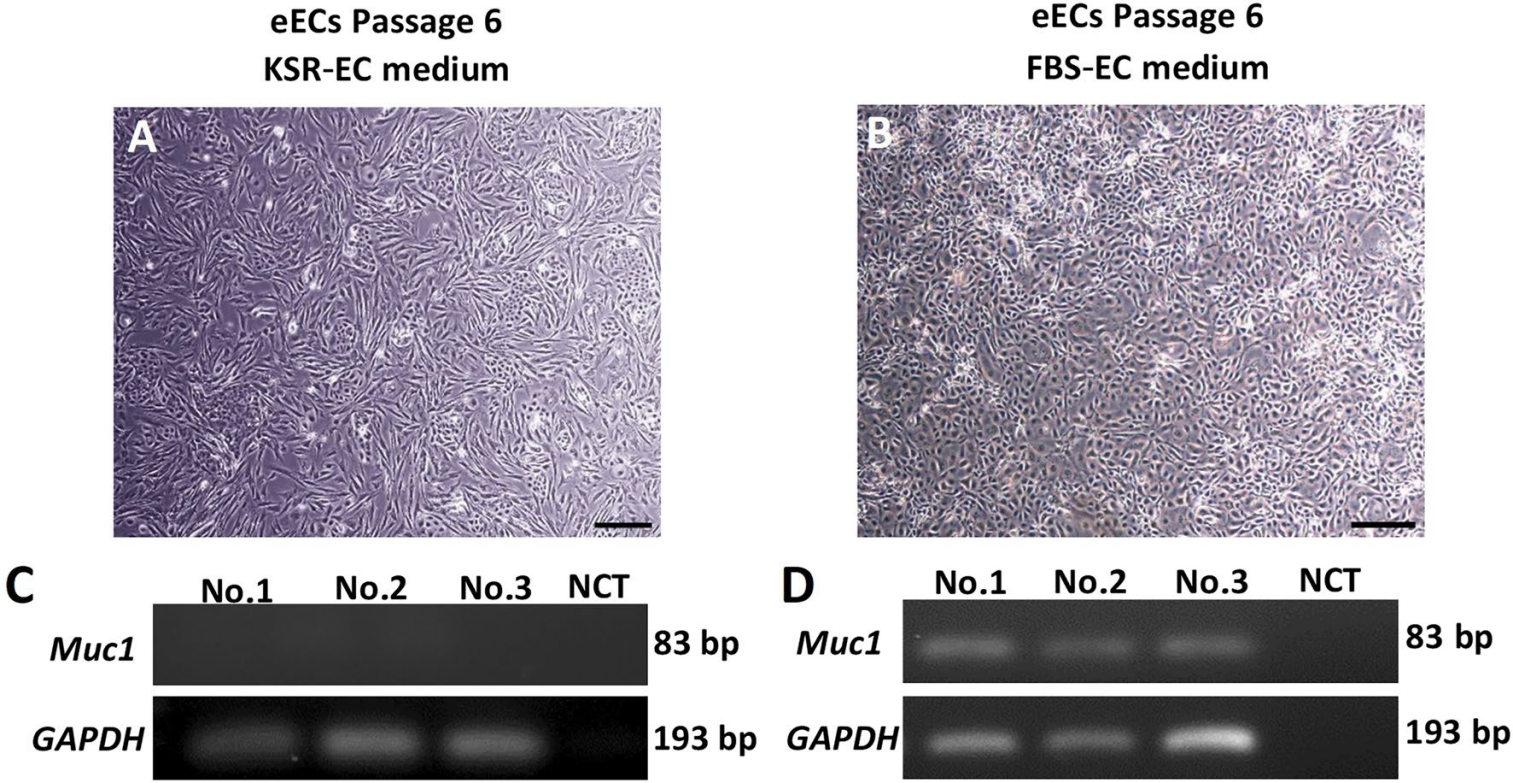
Morphology of eECs from passage 6 in; (**A**) KSR-EC medium, and (**B**) FBS-EC medium. (**C**) *Muc1* gene expression was not detected in eECs cultured in KSR-EC medium. (**D**) In contrast, eECs cultured in FBS-EC medium did show *Muc1* expression. Scale bar: 300 µm. (Uncropped conventional PCR gels were shown in the Supplementary Fig. 2).

While 10 µM of Y-27632 proved to be an effective supplement for enhancing eEC number and viability, it was evident that long term eECs quality could not be improved solely by treatment with a ROCK inhibitor. In particular, conventional PCR indicated that gene expression for the epithelial specific mucin 1 (*Muc1*) could not be sustained beyond Passage 6 when eECs were cultured in defined culture medium (10% KSR-EC media + 10 µM Y27632) (Fig. 4A,C). In contrast, culture medium containing FBS (10% FBS-EC) maintained eEC function, in terms of *Muc1* gene expression, beyond passage 6 (Fig. 4B,D).

Therefore, FBS-EC medium supplemented with 10 µM of Y-27632 was selected as the preferred culture condition for subsequent experiments.

### Reconstruction of equine in vitro 3D-ET

#### Morphological characterization

**Figure 5:**
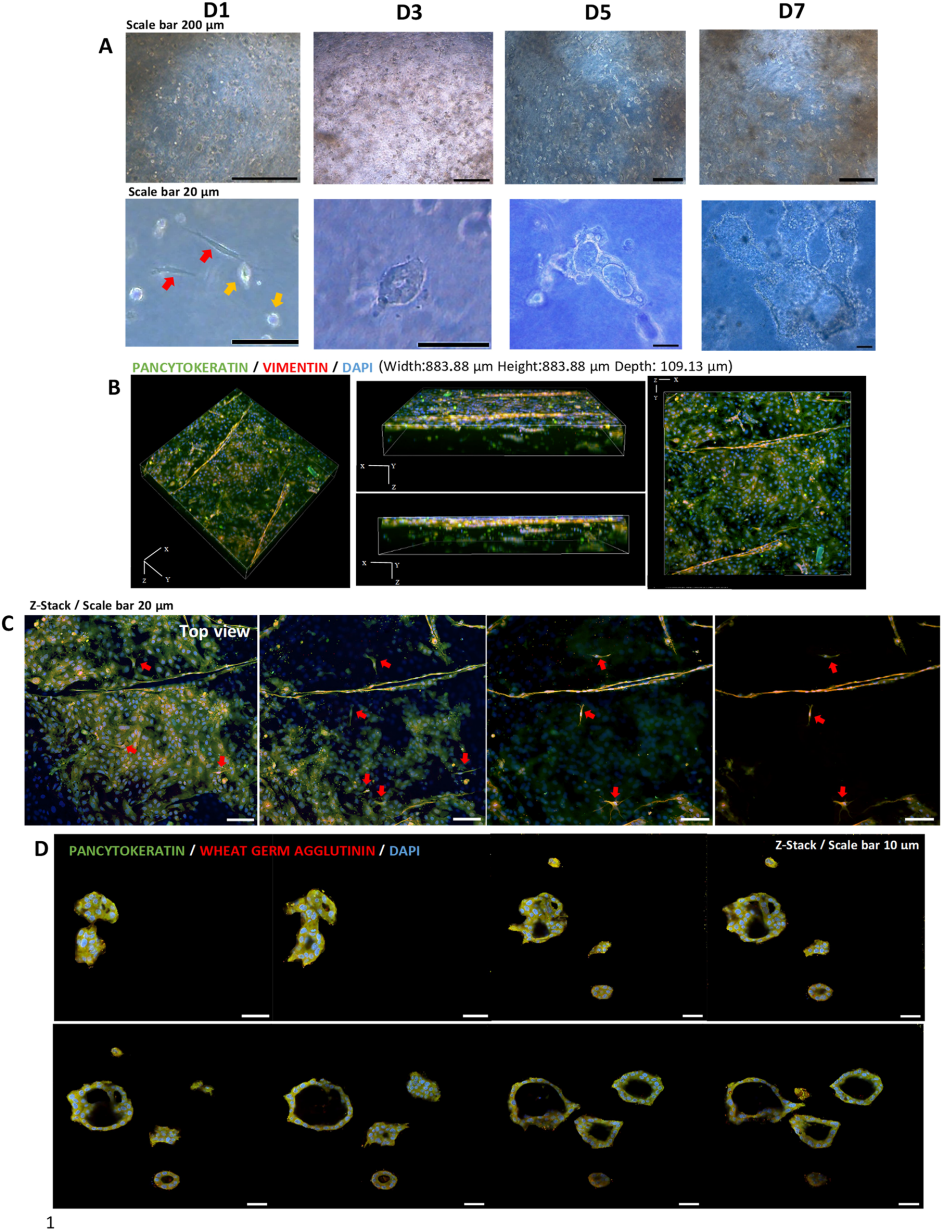
(**A**) The progression of an in vitro 3D-ET from Day 1 to Day 7 of culture. At D1, eMSCs (red arrow) exhibit a spindle-like shape in the collagen scaffold, while eECs display a round cellular shape (yellow arrow). By the following day, eECs have undergone cell division and differentiate into gland-like structures. By Days 5–10, these glands develop into tubular structures. (**B**) Confocal microscopy Z-stack panoramic view of the 3D structure of the 3D-ET. (**C**) The z-stack showing the various layers of the 3D-ET from left to right. The top view illustrates eECs positioned at the uppermost layer of the endometrial tissue. Red arrows highlight eMSCs in each layer of the 3D-ET, displaying a three-dimensional spindle shape with elongated processes. (**D**) Confocal microscopy shows that these ‘glands’ were hollow.

After 24 h of culture, the eMSCs displayed an elongated, spindle-shaped morphology with 3D processes, whereas the eECs appeared as single round cells that subsequently formed colonies. Within 2 to 3 days, the eECs underwent a transformation, developing into uniformly distributed spheroids, which subsequently evolved into gland-like structures, and even took on tubular configurations, between days 5 and 7. This in vitro 3D-ET could be maintained for approximately 14 days, with complete development of gland-like structures occurring around days 7 to 10. Results are illustrated in Fig. 5A.

**Figure 6:**
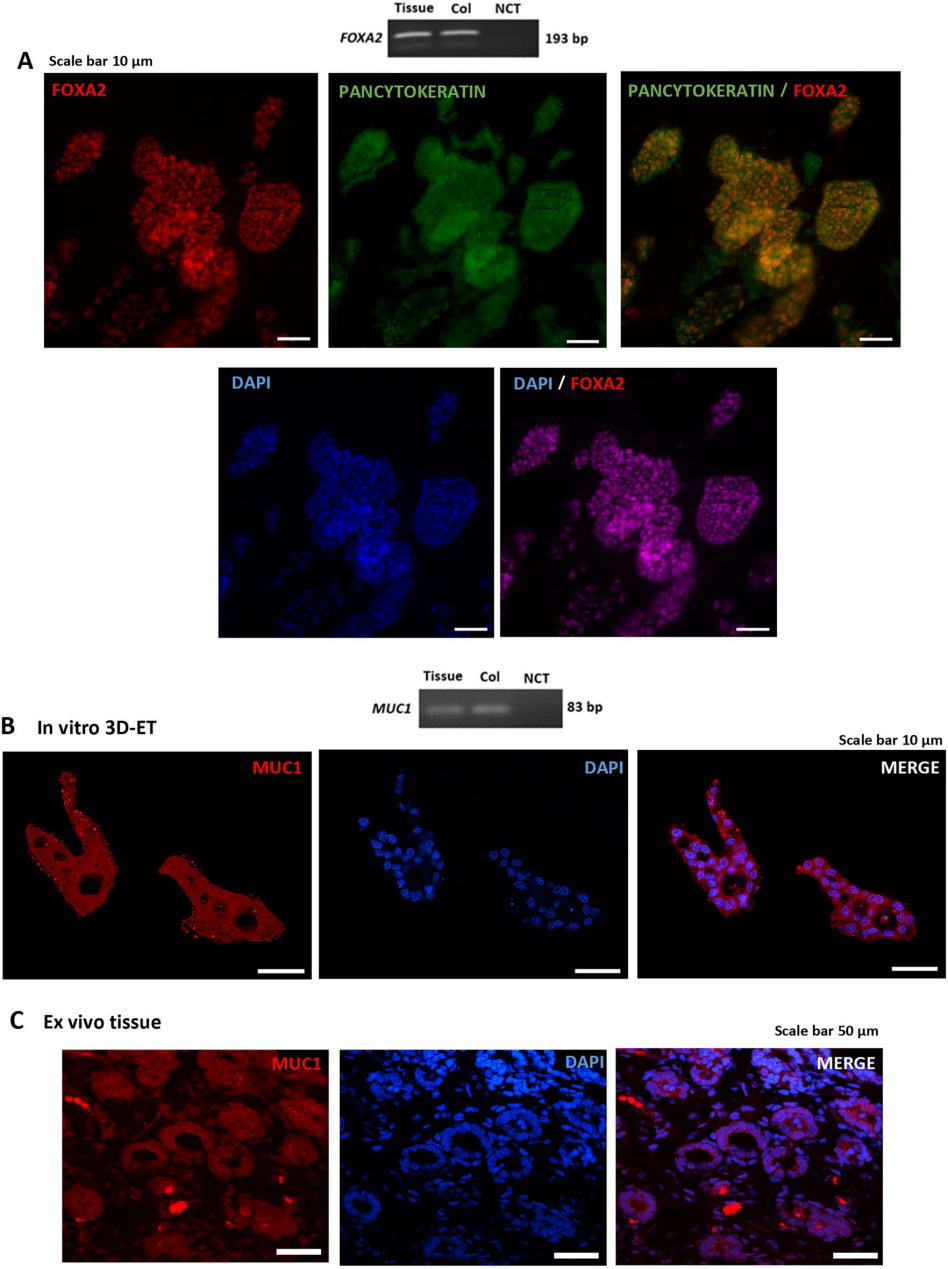
(**A**) Expression of the *FOXA2* gene in in vivo endometrial tissue and in the in vitro 3D-ET. FOXA2 protein was localized to the nucleus of the in vitro gland-like structures. (**B**) *Muc1* gene expression in in vitro 3D-ET and (**C**) in vivo tissue with MUC1 protein expression in the cytoplasm of the cells lining the gland. (Uncropped conventional PCR gels were shown in the Supplementary Fig. 5).

Immunofluorescence and confocal microscopy showed positive Pan Cytokeratin staining in the eECs making up the ‘glands’, whereas Vimentin staining was evident in the freely located eMSCs surrounding the glands. Wheat germ agglutinin staining (red) delineated the cell membrane and gland boundary (Fig. 5B,C). Z-stacks created via confocal microscopy offered insights into the gland’s 3D morphology, notably its hollow core surrounded by a monolayer of epithelial cells (Fig. 5D). Additionally, FOXA2 expression was observed in the nuclei of the cells lining the gland-like structure, and the *Foxa2* gene was detected in the in vitro 3D-ET (Fig. 6A).

#### Functional characterization

The *Muc1* gene was expressed in the in vitro 3D-ET, as confirmed by conventional PCR. Similarly, the expression of the secretory protein MUC1 was shown by red staining on the surface of the epithelial cells within the gland-like structures in both in vitro 3D-ET (Fig. 6B) and ex vivo tissue (Fig. 6C), as imaged via confocal microscopy.

**Figure 7:**
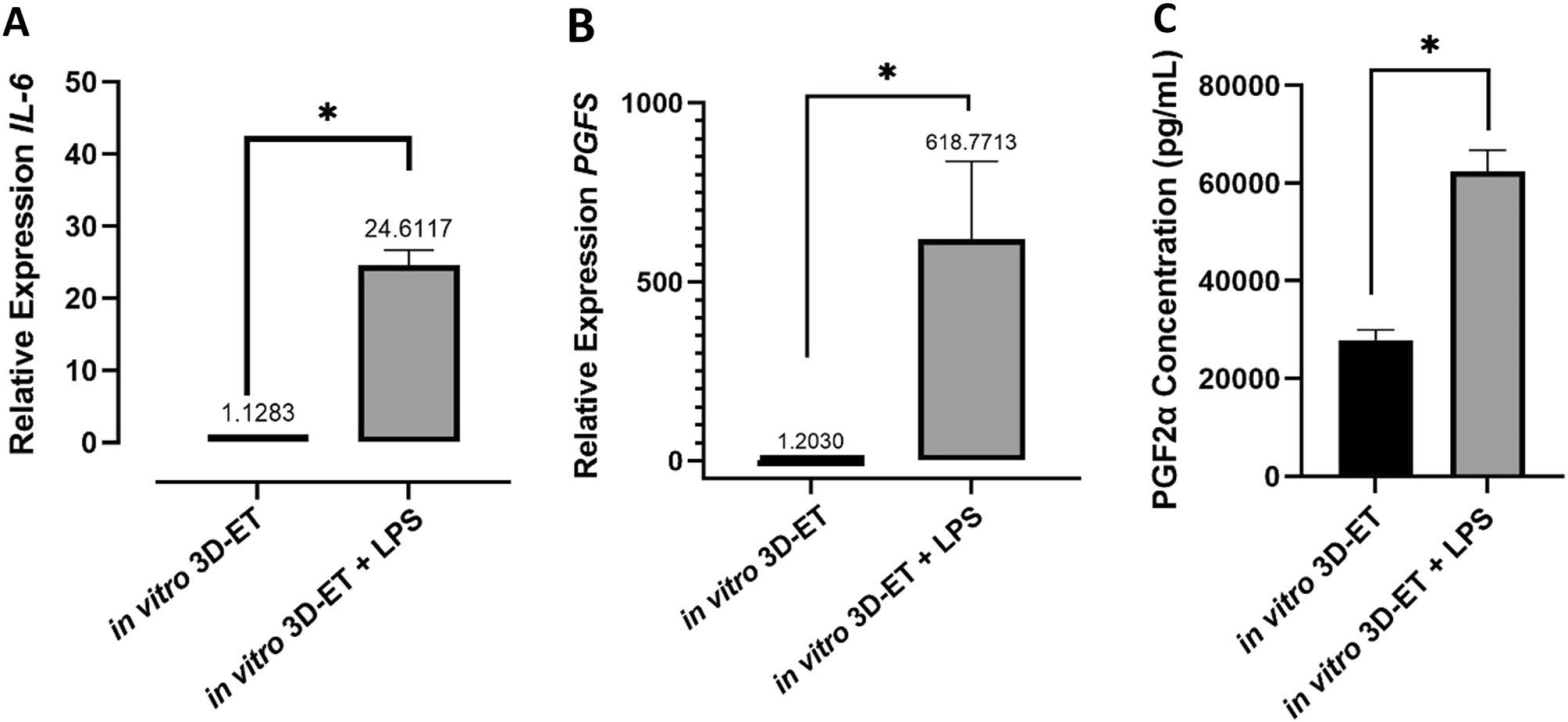
Relative expression of the (**A**) *IL6* and (**B**) *PGFS* genes in LPS-treated group versus control in vitro 3D-ET. (**C**) Secretion of PGF2α was significantly higher in the LPS-treated group (P < 0.01).

The relative expression of the *IL6* and *PGFS* genes in the LPS-treated constructs was significantly higher than in the control (P < 0.01: Fig. 7A,B). Additionally, PGF2α secretion in the LPS-treated group was significantly higher than in the control group (P < 0.001: Fig. 7C). The ELISA for IL6 was semiquantitative and the results revealed that, in the control group, IL6 secretion was detected only at levels < 5.13 pg/ml (100%). In contrast, 66.67% of constructs in the LPS-treated had IL6 secretion < 5.13 pg/ml, and 33.33% had IL6 secretion at 10.36 pg/ml (see Table 3).

**Table 3:** IL6 secretion measured using a semi-quantitative ELISA kit.

ELISA	Number (%) secreting IL6	
	< 5.13 pg/ml	10.36 pg/ml
In vitro 3D-ET	18/18 (100%)	0/18 (0%)
In vitro 3D-ET + LPS	12/18 (66.67%)*	6/18 (33.33%)**

*,**Within a column, rates of IL6 secretion with different superscripts differ significantly (P < 0.01) by Chi-square (Fisher’s exact test).

## Discussion

This study investigated the isolation and optimization of culture systems for eECs and eMSCs, and emphasized the significance of small molecules, notably a Rock inhibitor, in enhancing the activity of some cell types. The study introduces the first de novo synthesized 3D equine endometrium model using a collagen base membrane and optimal culture conditions. At first, our eMSCs adhered to a plastic plate and expressed some MSC global genes such as *CD29*, *CD90*, and *CD44* similar to a previous report^25^. More importantly, these isolated eMSCs could differentiate into mesodermal lineage (osteogenic and adipogenic lineages). Although minimal criteria for MSC characterization have been proposed in humans^26^, the full characterization of MSCs has yet to be fully established in equines. Further investigation for chondrogenic lineage is needed for a comprehensive understanding of eMSCs differentiation potential. Indeed, a mixed population of other cell types such as fibroblasts or other stromal cells is also possible. Rho-associated protein kinase (ROCK) is known to play a crucial role in various cell functions through the Rho/ROCK signaling pathway, specifically influencing cytoskeletal dynamics and contraction. This study demonstrated the presence of elements of the Rho/ROCK/LIM Kinase signaling pathway within eECs and revealed that the ROCK inhibitor, Y-27632, significantly reduced the doubling time of eECs, indicating increased cell proliferation and viability, aligning with findings reported for other epithelial cell types^32–34^. While 10 µM Y-27632 emerged as the best concentration for stimulating eECs in terms of proliferative activity and apoptosis reduction in the current study a higher dose (20 µM Y-27632) was reported to significantly increase S-phase survival in limbal epithelial cells^35^. Further study should determine the optimal concentration of ROCK inhibitor for culturing eECs, since it appears that different cell types require different specific concentrations of ROCK inhibitor. In response to H_2_O_2_ exposure, the eECs underwent apoptosis and necrosis, with a higher percentage of viable cells remaining in medium containing 10 µM Y-27632. While the culture in the presence of 10 µM Y-27632 was associated with an increased expression of the proapoptotic gene, BAX*,* there was a marked concomitant rise in expression of the anti-apoptotic, *BCL2*, resulting in an overall reduction in the *BAX*: *BCL2* ratio (i.e., anti-apoptotic response). Similarly, HeLa Clone9 cells^13^ and cortical neurons^36^ exhibit a reduced early apoptotic blebbing when preincubated with 10 µM Y-27632. This anti-apoptotic effect has been suggested to involve the p38-MAPK, ROCK-I, and MLCK pathways. On the other hand, the current study confirmed the expression of elements of the Rho/LIMKinase pathways in eECs. Further study of the function of the various kinases would be of interest because the functions of LIMK1 and *LIMK2* appear to be cell type specific. For example, LIMK2 activation by RhoA depended on ROCK activity, whereas Rac1-dependent LIMK1 activation was unaffected by ROCK^37^.

This study additionally found that early passages of eECs retained *Muc1* gene expression in both FBS-EC and KSR-EC media. However, *Muc1* expression was absent as early as passage 6 in the KSR-EC medium. This suggests that KSR-EC may only partially support eEC activity and function. We hypothesize that *Muc1* expression in eECs requires some additional factors, such as sex steroid hormones or growth factors, present in the FBS included in the FBS-EC medium^38,39^. However, it is essential to consider the potential impact of the variations of batch-to-batch fetal bovine serum (FBS) as an animal-derived product, on the expression of *Muc1*.

The successful de novo reconstruction of equine endometrial tissue provides a new perspective on equine uterine adenogenesis; FOXA2 has been identified as a key intrinsic factor crucial for gland development and, therefore, an important fertility marker in species such as the rat, man, pig, and sheep^40–42^. In addition, equine endometrium requires collagen types I, III, IV, and laminin to establish a microenvironment and support the cellular processes required for uterine gland formation^43,44^. In the current study, the eECs and eMSCs were cultured in a collagen BM scaffold, known for its diverse protein content (approximately 20 proteins, such as laminin 111, collagen IV, perlecan, nidogen 1, nidogens, and two additional proteoglycans, agrin and collagen XVIII)^44,45^. This provides biochemical and biomechanical properties that facilitate the establishment of an appropriate microenvironment for differentiation and communication between eECs and eMSCs during adenogenesis^43–45^. Additionally, Y-27632 may be a pivotal factor, playing a key role in supporting the initial differentiation and proliferation of the eECs.

The equine 3D-ET model demonstrated an in vivo-like uterine structure by maintaining structural polarity, differentiated function, and gland development similar to in vivo conditions. Although we did not compare to other methods, the 3D-ET’s functionality aligns with previous results of tissue explant and 2D conventional culture methods^46^. Upon LPS stimulation, the 3D-ET model responded by upregulating genes associated with inflammatory pathways (*IL6* and *PGFS*) and their secretory products, suggesting a functional innate immune system. However, limitations such as culture time constraints, scaffold instability, batch-to-batch variation of the collagen ECM, and aging of eECs hinder comprehensive exploration of long-term physiology, including endometrial gland tubulogenesis (post-natal 10–14 days)^40^, and pathological aspects like endometrosis^47,48^. In addition, the stromal and epithelial ratio for uterine adenogenesis may be crucial^49^, Our preliminary investigation revealed no discernible variation in either morphology or quantity of gland development when employing stromal: epithelial cell ratios of 1:1, 1:5 and 1:10 in the de novo reconstructed endometrial tissue (see Supplementary Fig. S1). The 1:1 ratio was selected for this study as it seemed more appropriate for prolonged culture than other ratios, which showed an apoptosis-like phenomenon between days 7 and 10 of culture. However, several studies on endometrial 3D co-culture propose diverse stromal: epithelial ratios dependent on the species and experimental design^50–52^. Nevertheless, studies on the effects of long term culture with different ratios of the cells on uterine gland development and function will be important for further optimizing the structure and properties of the 3D equine endometrial model. Moreover, advancing the culture system through the integration of bioengineering principles is critical for maintaining long-term culture and expanding the exploration of various aspects of physiology, pathology, and pharmacology. In conclusion, this study successfully established an in vitro 3D endometrial tissue reconstruction that closely mimics the structure and shows a number of specific functions of in vivo endometrial tissue. This in vitro model has the potential to advance research on various aspects of normal uterine physiology and pathological conditions in the horse and may be a useful comparative model for other species.

### Supplementary Information

Supplementary Figures.

Supplementary Information 1.

Supplementary Information 2.

## Data Availability

All data generated or analyzed during this study are included in this published article (and its Supplementary Information files) and the datasets used and/or analysed during the current study available from the corresponding author on reasonable request.
