# Supplementary material for: De novo reconstruction of a functional in vivo-like equine endometrium using collagen-based tissue engineering
Source: Sci Rep. 2024 Apr 19;14:9012. doi: 10.1038/s41598-024-59471-z (PMC11031578; doi:10.1038/s41598-024-59471-z)
Supplement: Supplementary file 1 — Supplementary Figures. [file 41598_2024_59471_MOESM1_ESM.pdf]

Supplementary Figures

De novo reconstruction of a functional *in vivo*-like equine endometrium using collagen-based tissue engineering

Sawita Santiviparat[a,b,c], Theerawat SwangchanU-thai[a,b], Tom A.E. Stout[d], Supranee Buranapraditkun[e,f,g], Piyathip Setthawong[h], Teeanutree Taephatthanasagon[i,j], Watchareewan Rodprasert[i,j], Chenphop Sawangmake[i,j,k,l], *Theerawat Tharasanit[a,b,c]

[a] Department of Obstetrics, Gynecology and Reproduction, Faculty of Veterinary Science Chulalongkorn University , Bangkok, Thailand

[b] CU-Animal Fertility Research Unit, Chulalongkorn University, Bangkok, Thailand

[c] Veterinary Clinical Stem Cells and Bioengineering Research Unit, Chulalongkorn University, Bangkok, Thailand

[d] Department of Clinical Sciences, Utrecht University, Utrecht, The Netherlands.

[e] Division of Allergy and Clinical Immunology, Department of Medicine, King Chulalongkorn Memorial Hospital, Faculty of Medicine, Chulalongkorn University, Thai Red Cross Society, Bangkok 10330, Thailand

[f] Center of Excellence in Vaccine Research and Development (Chula Vaccine Research Center-Chula VRC), Faculty of Medicine, Chulalongkorn University, Bangkok 10330, Thailand

[g] Thai Pediatric Gastroenterology, Hepatology and Immunology (TPGHAI) Research Unit, King Chulalongkorn Memorial Hospital, Faculty of Medicine, Chulalongkorn University, The Thai Red Cross Society, Bangkok 10330, Thailand,

[h] Department of Physiology, Faculty of Veterinary Medicine, Kasetsart University, Bangkok, Thailand

[i] Veterinary Pharmacology and Stem Cell Research Laboratory, Veterinary Stem Cell and Bioengineering Innovation Center (VSCBIC), Faculty of Veterinary Science, Chulalongkorn University, Bangkok, Thailand

[j] Veterinary Systems Pharmacology Center (VSPC), Faculty of Veterinary Science, Chulalongkorn University, Bangkok, Thailand

[k] Department of Pharmacology, Faculty of Veterinary Science, Chulalongkorn University, bangkok, Thailand

[l] Center of Excellence in Regenerative Dentistry, Faculty of Dentistry, Chulalongkorn University, Bangkok, Thailand,

*Corresponding author. E-mail address: Theerawat.t@chula.ac.th (T.Tharasanit)

The preliminary ratio of stromal: epithelial cells in the *de novo* reconstructed 3D-ET

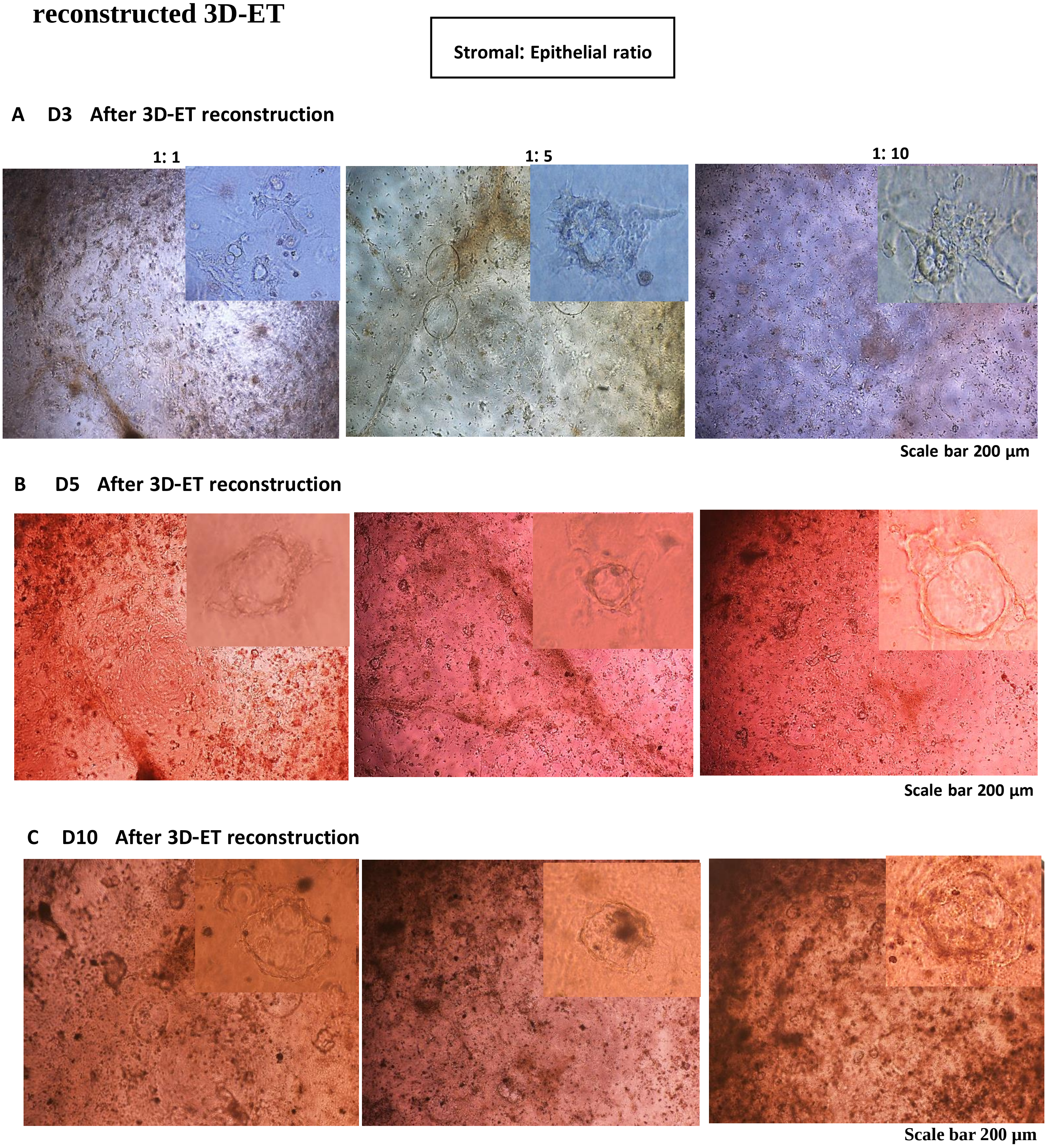

Supplementary Figure 1: In the preliminary 3D-ET reconstruction **A**) the ratio between stromal and epithelial cells was observed at D3 after seeding. At this stage, 3d-ETs from all ratios began illustrating gland-like structures. **B**) The gland-like structures exhibited continued growth, eventually developing into hollow structures. **C**) At D10 of culture, all 3D-ET initial cell ratios exhibited pigmentation in the glands, indicating glandular apoptosis. However, at seeding ratios of 1:5 and 1:10, the apoptotic effect was more pronounced than at the 1:1 ratio.

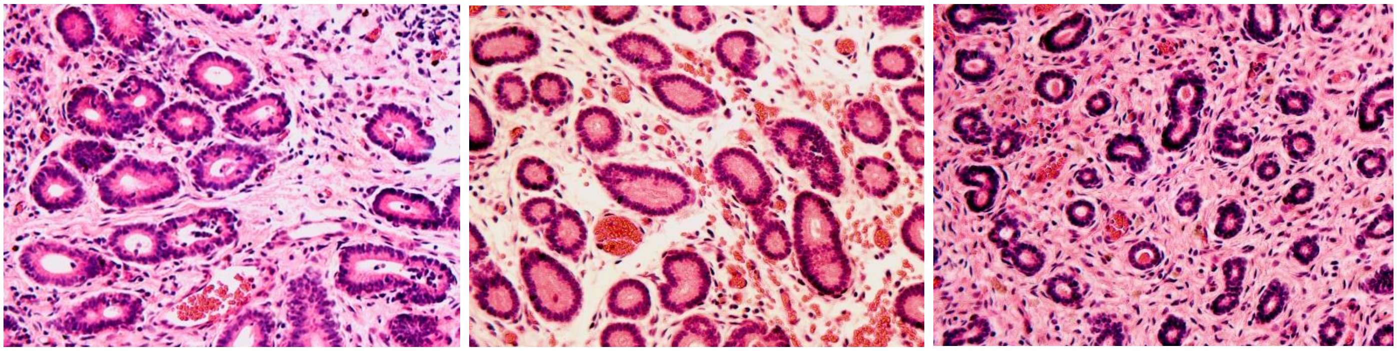

Supplementary Figure 2: displays examples of endometrial tissue post-biopsy specifically selected as exhibiting normal healthy endometrium (grade 1) according to Kenney, 1986. The figures illustrate either a normal endometrium or mild focal inflammation or fibrosis.

interleukin-4 precursor [Equus caballus]

Sequence ID: NP_001075988.1 Length: **137** Number of Matches: **1**

See 5 more title(s) See all Identical Proteins(IPG)

Range 1: 1 to 137 GenPept Graphics ▼ Next Match ▲ Previous Match

Score	Expect	Method	Identities	Positives	Gaps
145 bits(366)	1e-50	Compositional matrix adjust.	85/154(55%)	98/154(63%)	20/154(12%)

```
Query  1    MGLTSQLLPPLFFLLACAGNFVHGHKCDITLQEIIKTLNSLTE--QKTLCTELTVTDIFA  5[illegible]
            MGLT QL+P L  LLAC  NF+ G K DITLQEIIKTLN+LT+   K  C ELTV D FA
Sbjct  1    MGLTYQLIPALVCLLACTSNFIQGCKYDITLQEIIKTLNNLTDGKGKNSCMELTVADAFA  6[illegible]

Query  59   ASKNTTEKETFCRAATVLRQFYSHHEKDTRCLGATAQQFHRHKQLIR-FLKRLDRNLWGL  1[illegible]
              KNT  KE  CRAA VL+Q Y  H+                + LI+  L  LDRNL G+
Sbjct  61   GPKNTDGKE-ICRAAKVLQQLYKRHD----------------RSLIKECLSGLDRNLKGM  1[illegible]

Query  118  AGLNSCPVKEANQSTLENFLERLKTIMREKYSKC  151
            A    C V EA +STL++FLERLKTIM+EKYSKC
Sbjct  104  ANGTCCTVNEAKKSTLKDFLERLKTIMKEKYSKC  137
```

interferon gamma precursor [Equus caballus]

Sequence ID: NP_001075418.1 Length: **166** Number of Matches: **1**

See 4 more title(s) See all Identical Proteins(IPG)

Range 1: 1 to 166 GenPept Graphics ▼ Next Match ▲ Previous Match

Score	Expect	Method	Identities	Positives	Gaps
224 bits(571)	4e-81	Compositional matrix adjust.	112/166(67%)	130/166(78%)	0/166(0%)

```
Query  1    MKYTSYILAFQLCIVLGSLGCYCQDPYVKEAENLKKYFNAGHSDVADNGTLFLGILKNWK  6[illegible]
            M YTS+ILAFQLC +LGS   YCQ  + KE ENLK+YFNA + DV D G LFL ILKNWK
Sbjct  1    MNYTSFILAFQLCAILGSSTYYCQAAFFKEIENLKEYFNASNPDVGDGGPLFLDILKNWK  6[illegible]

Query  61   EESDRKIMQSQIVSFYFKLFKNFKDDQSIQKSVETIKEDMNVKFFNSNKKKRDDFEKLTN  1[illegible]
            E+SD+KI+QSQIVSFYFKLF+N KD+Q IQKS++TIKED+ VKFFNS+  K +DF+KL
Sbjct  61   EDSDKKIIQSQIVSFYFKLFENLKDNQVIQKSMDTIKEDLFVKFFNSSTSKLEDFQKLIQ  1[illegible]

Query  121  YSVTDLNVQRKAIHELIQVMAELSPAAKTGKRKRSQMLFRGRRASQ  166
              V DL VQRKAI ELI+VM +LSP A   KRKRSQ  FRGRRA Q
Sbjct  121  IPVNDLKVQRKAISELIKVMNDLSPKANLRKRKRSQNPFRGRRALQ  166
```

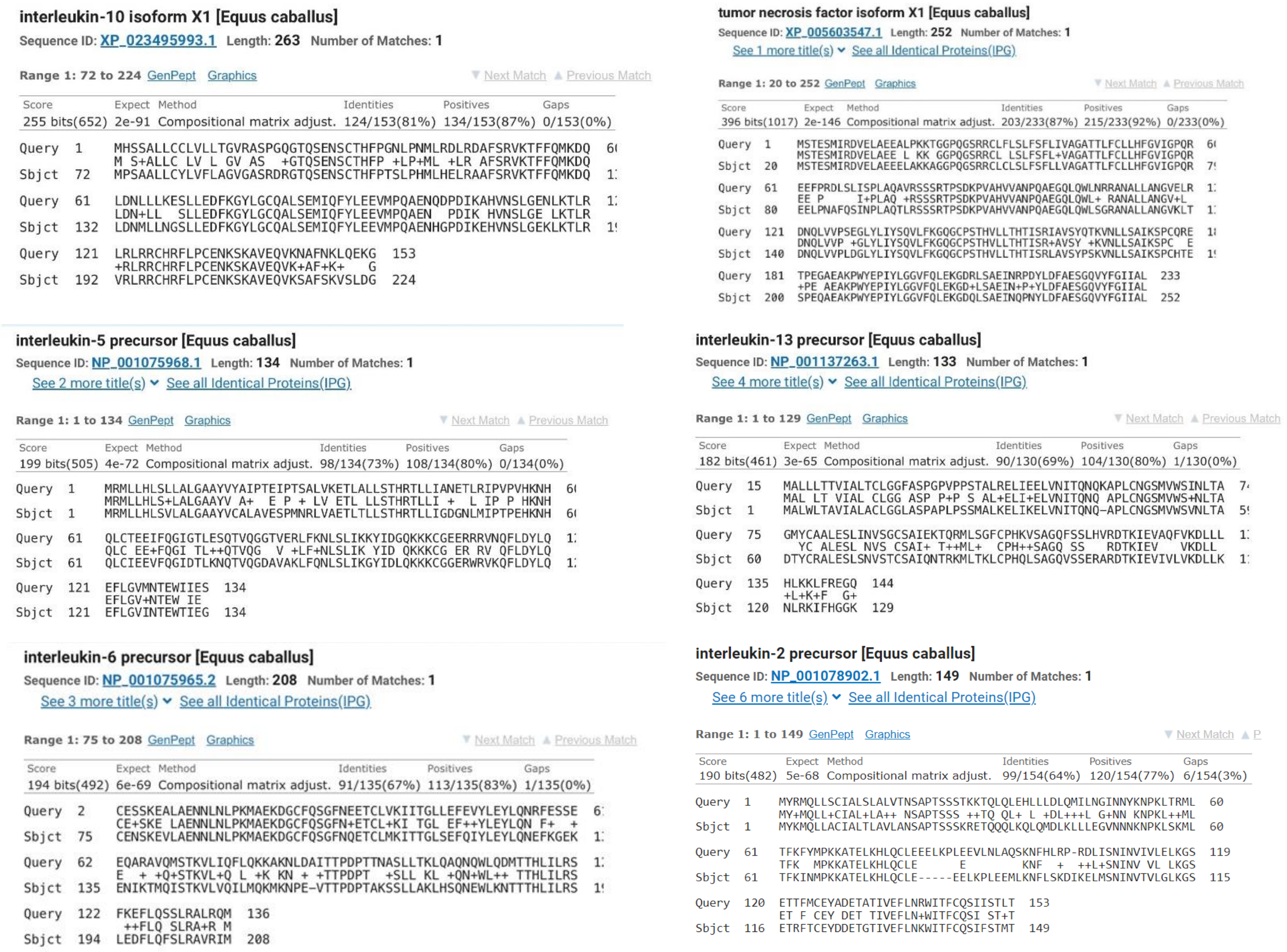

Supplementary Figure 3: The homology of eight inflammatory cytokines in response to LPS induction was investigated. Utilizing the Human Th Cytokine Panel 13-plex assay from Biolegend, USA, and employing flow cytometry analysis on a BD FACS Calibur instrument from Becton Dickinson, USA, we found interesting parallels between equine and human sequences. Specifically, the percent homology was highest for IFNY (67%), IL13 (69%), IL5 (73%), IL10 (81%), IL6 (67%), and TNF-α (87%), IL2 (64%)consistent with previous observations by Scheerlinck in 1999. (It's noteworthy that >60% homology suggests potential cross-reactivity with cytokines). While our study encompassed the examination of eight cytokines, particular emphasis was placed on IL6, which emerged as a pivotal cytokine of interest.
