# Supplementary material for: De novo reconstruction of a functional in vivo-like equine endometrium using collagen-based tissue engineering
Source: Sci Rep. 2024 Apr 19;14:9012. doi: 10.1038/s41598-024-59471-z (PMC11031578; doi:10.1038/s41598-024-59471-z)
Supplement: Supplementary file 2 — Supplementary Information 1. [file 41598_2024_59471_MOESM2_ESM.pdf]

Supplementary Materials and Methods

De novo reconstruction of a functional *in vivo*-like equine endometrium using collagen-based tissue engineering

Sawita Santiviparat[a,b,c], Theerawat SwangchanU-thai[a,b], Tom A.E. Stout[d], Supranee Buranapraditkun[e,f,g], Piyathip Setthawong[h], Teeanutree Taephatthanasagon[i,j], Watchareewan Rodprasert[i,j], Chenphop Sawangmake[i,j,k,l], *Theerawat Tharasanit[a,b,c]

[a] Department of Obstetrics, Gynecology and Reproduction, Faculty of Veterinary Science Chulalongkorn University , Bangkok, Thailand

[b] CU-Animal Fertility Research Unit, Chulalongkorn University, Bangkok, Thailand

[c] Veterinary Clinical Stem Cells and Bioengineering Research Unit, Chulalongkorn University, Bangkok, Thailand

[d] Department of Clinical Sciences, Utrecht University, Utrecht, The Netherlands.

[e] Division of Allergy and Clinical Immunology, Department of Medicine, King Chulalongkorn Memorial Hospital, Faculty of Medicine, Chulalongkorn University, Thai Red Cross Society, Bangkok 10330, Thailand

[f] Center of Excellence in Vaccine Research and Development (Chula Vaccine Research Center-Chula VRC), Faculty of Medicine, Chulalongkorn University, Bangkok 10330, Thailand

[g] Thai Pediatric Gastroenterology, Hepatology and Immunology (TPGHAI) Research Unit, King Chulalongkorn Memorial Hospital, Faculty of Medicine, Chulalongkorn University, The Thai Red Cross Society, Bangkok 10330, Thailand,

[h] Department of Physiology, Faculty of Veterinary Medicine, Kasetsart University, Bangkok, Thailand

[i] Veterinary Pharmacology and Stem Cell Research Laboratory, Veterinary Stem Cell and Bioengineering Innovation Center (VSCBIC), Faculty of Veterinary Science, Chulalongkorn University, Bangkok, Thailand

[j] Veterinary Systems Pharmacology Center (VSPC), Faculty of Veterinary Science, Chulalongkorn University, Bangkok, Thailand

[k]Department of Pharmacology, Faculty of Veterinary Science, Chulalongkorn University, bangkok, Thailand

[l] Center of Excellence in Regenerative Dentistry, Faculty of Dentistry, Chulalongkorn University, Bangkok, Thailand,

*Corresponding author. E-mail address: Theerawat.t@chula.ac.th (T.Tharasanit)

2.1.3 Characterization of the eECs and eMSCs

Immunofluorescence: The eECs and eMSCs were cultured on coverslips coated with 0.1% (w/v) gelatin. At 50% confluence, the cells were fixed with 4% (w/v) paraformaldehyde and permeabilized using 0.1 M Glycine and 0.1% (v/v) TritonX-100, respectively. The eECs and eMSCs were then incubated at 4° C overnight with primary antibody. Thereafter, the cells were washed twice with 1xPBS and incubated with fluorescence-labeled secondary antibody for 1 h at 37° C. The dilution of primary and secondary antibodies is shown in Table 2. Nuclei were stained with 4’6-diamidino-2-phenylindole (DAPI) (Sigma). Finally, an antifade mounting medium (VECTASHIELD®) was added to the coverslip for protection against photobleaching.

Flow cytometry: To assess purity, the eECs from both EC media conditions were digested with 0.25% (w/v) trypsin-EDTA and fluorescently stained as described previously. The expression of green fluorescent protein (Pan Cytokeratin) was analyzed by flow cytometer (BD FACS Calibur, Becton Dickinson, USA). The quantification of green-positive cells facilitated evaluation of eEC purity.

***In vitro* osteogenic and adipogenic differentiation:** To induce osteogenic differentiation, the eMSCs were seeded in triplicate in 6-well plates (20,000 cells/cm^2) and cultured in MSC media for 2-3 days until they reached confluency. Subsequently, modified osteogenic differentiation media, based on Rink et al. (2017), were introduced to the well and incubated in humidified conditions at 37°C, 5% CO_2. The media were changed every 2-3 days until day 14. The cells were then divided into 2 portions: one portion was collected for conventional PCR, targeting the specific gene *COL1A1* (Primer sequence illustrated in Table 1), while the other portion was fixed in 10% formalin and stained with alizarin red (A5533-56, Sigma-Aldrich) for 10-15 min to visualize extracellular calcium deposits using light microscopy. In addition, cellular activity was evaluated using an Alkaline Phosphatase kit (Sigma-Aldrich), with the staining protocol following the manufacturer's instructions.

To induce adipogenic differentiation, eMSCs (n=3) were cultivated in triplicate within 24-well plates as described previously. After reaching confluency, the culture medium was replaced with a Human adipogenic medium (MesenCult™), and cells were maintained under these conditions for 7 days. Following this period, cells were fixed using 4% paraformaldehyde and subsequently stained with oil red O (Sigma Aldrich) for 15 min to visualize lipid accumulation. Concurrently, another set of cells was trypsinized and stored at -80°C for subsequent detection of specific gene (*LPL*) expression by conventional PCR using primers outlined in Table 1.

Primer design, RNA extraction, and conventional PCR: Primer sequences were designed using Primer3 Web-based software based on high-quality nucleotide sequences derived from the coding domain sequence of genes in the National Center for Biotechnology Information (NCBI; http://www.ncbi.nlm.nih.gov/) and Rink *et al.* (2017) as illustrated in Table 1. The specificity, compatibility, and potential dimers of the designed primer were determined by NCBI Primer-BLAST and OligoAnalyzer version 3.1 software (http://eu.idtdna.com/analyzer/Applications/OligoAnalyzer/) before being synthesized (Eurofins MWG Operon). Reference genes (*GAPDH*), an Epithelial marker (*MUC1*), MSC markers (*CD29*, *CD44*, *CD90*), an osteogenic marker (*COL1A1*), and an adipogenic marker (*LPL*) were studied. The Rneasy Minikit (Qiagen, Hilden, Germany) was employed for total RNA extraction from cell pellet samples. The extracted mRNA quality was assessed using NanoDrop™ 2000 (Wilmington, DE, USA), ensuring an A260/A280 ratio of 1.9 to 2.1 before proceeding to RT-PCR. Removal of residual DNA involved treatment with DNAseI (RQI Rnase-Free Dnase, Promega WI, USA). The ImprompT II ™ Reverse Transcription System (Promega, Madison, WI, USA) facilitated cDNA synthesis in accordance with the manufacturer's instructions. The total amount RNA is 1000 ng for synthesizing cDNA. PCR was performed using goTaq® Green Master Mix (Promega, Madison, WI, USA) involving initial denaturation (2 min, 95°C), 30-40 amplification cycles with specific primer annealing temperatures (30 sec), and a final extension step (2 min, 72°C). Resultant PCR products were separated using 1% agarose gel with 1x Tris/Borate/EDTA buffer (TBE buffer, pH 8.0, 2 mM EDTA, 90 mM Tris, 90 mM boric acid). Gel visualization employed Redsafe Nucleic Acid Staining Solution (iNtRON Biotechnology, Gyeonggi-do, Korea) and UV light via a Gel documentation system (Syngene, Cambridge, UK).

2.3.3 Characterization of *in vitro* 3D reconstructed endometrial tissue

Immunofluorescent and confocal microscopy: Characterization of secretion function of the endometrial gland-like structure was carried out by staining with a MUC1 primary antibody (sc-7313, Santa Cruz Biotechnology), with a staining protocol adapted from Piña *et al*. (2022). After fixation, the *in vitro* 3D-ET underwent two 3 min washes with 0.5% Tween 20 in 1x PBS. Non-specific binding was blocked using 2% bovine serum albumin (BSA), 50 mM Glycine, 0.05% Tween 20, 0.1% Triton X, and 0.01% BSA. Primary antibody was prepared at a 1:50 dilution in 1x PBS, 10 mM Glycine, 0.05% Tween 20, 0.1% Triton X, and 0.1% H_2O_2 and incubated overnight at 4°C. The secondary antibody (as listed in Table 2) was diluted in 0.1% Tween 20 in PBS and incubated for 30 minutes at room temperature. DAPI was used to stain the nuclei. Post-staining, an anti-fade reagent was applied to the *in vitro* tissue, before covering it with a cover glass and mounting using a mounting solution. The overall structure was visualized using an immunofluorescent microscope, while a confocal microscope (AX/AXR with NSPARC, Nikon Japan) was used for 3D structure assessment.

ELISA: All media supernatant from the *in vitro* 3D-ET system were used to determine IL6 levels in duplicate using the Human Th Cytokine Panel 13-plex, Biolegend, USA). Briefly, 25µL of assay buffer was added into each well. Then, 25 µl of diluted standard or serum was added to standard or sample wells. Next, 25 µL of mixed beads and 25 µL of detection antibodies were added into each well and incubated for 2 h at room temperature on an orbital plate shaker. After incubation, 25 µL of streptavidin-PE solution was added and then incubated for 30 min at room temperature on an orbital plate shaker. The plates were centrifuged at 1,000 rpm for 5 min. After decanting the liquid, the wells were washed more one time with wash buffer. Finally, 150 µL of wash buffer was added to all wells, which were shaken for 2-3 min before analysis by flow cytometry (BD FACS Calibur, Becton Dickinson, USA).
