# Supplementary material for: De novo reconstruction of a functional in vivo-like equine endometrium using collagen-based tissue engineering
Source: Sci Rep. 2024 Apr 19;14:9012. doi: 10.1038/s41598-024-59471-z (PMC11031578; doi:10.1038/s41598-024-59471-z)
Supplement: Supplementary file 3 — Supplementary Information 2. [file 41598_2024_59471_MOESM3_ESM.pdf]

# ***De novo*** **reconstruction of a functional *in vivo*-like equine endometrium using collagen-based tissue engineering**

Sawita Santiviparat[a,b,c], Theerawat SwangchanU-thai[a,b], Tom A.E. Stout[d], Supranee Buranapraditkun[e,f,g], Piyathip Setthawong[h], Teeanutree Taephatthanasagon[i,j], Watchareewan Rodprasert[i,j], Chenphop Sawangmake[i,j,k,l], *Theerawat Tharasanit[a,b,c]

[a] Department of Obstetrics, Gynecology and Reproduction, Faculty of Veterinary Science Chulalongkorn University, Bangkok, Thailand

[b] CU-Animal Fertility Research Unit, Chulalongkorn University, Bangkok, Thailand

[c] Veterinary Clinical Stem Cells and Bioengineering Research Unit, Chulalongkorn University, Bangkok, Thailand

[d] Department of Clinical Sciences, Utrecht University, Utrecht, The Netherlands.

[e] Division of Allergy and Clinical Immunology, Department of Medicine, King Chulalongkorn Memorial Hospital, Faculty of Medicine, Chulalongkorn University, Thai Red Cross Society, Bangkok 10330, Thailand

[f] Center of Excellence in Vaccine Research and Development (Chula Vaccine Research Center-Chula VRC), Faculty of Medicine, Chulalongkorn University, Bangkok 10330, Thailand

[g] Thai Pediatric Gastroenterology, Hepatology and Immunology (TPGHAI) Research Unit, King Chulalongkorn Memorial Hospital, Faculty of Medicine, Chulalongkorn University, The Thai Red Cross Society, Bangkok 10330, Thailand,

[h] Department of Physiology, Faculty of Veterinary Medicine, Kasetsart University, Bangkok, Thailand

[i] Veterinary Pharmacology and Stem Cell Research Laboratory, Veterinary Stem Cell and Bioengineering Innovation Center (VSCBIC), Faculty of Veterinary Science, Chulalongkorn University, Bangkok, Thailand

[j] Veterinary Systems Pharmacology Center (VSPC), Faculty of Veterinary Science, Chulalongkorn University, Bangkok, Thailand

[k] Department of Pharmacology, Faculty of Veterinary Science, Chulalongkorn University, bangkok, Thailand

[l] Center of Excellence in Regenerative Dentistry, Faculty of Dentistry, Chulalongkorn University, Bangkok, Thailand,

*Corresponding author. E-mail address: Theerawat.t@chula.ac.th (T.Tharasanit)

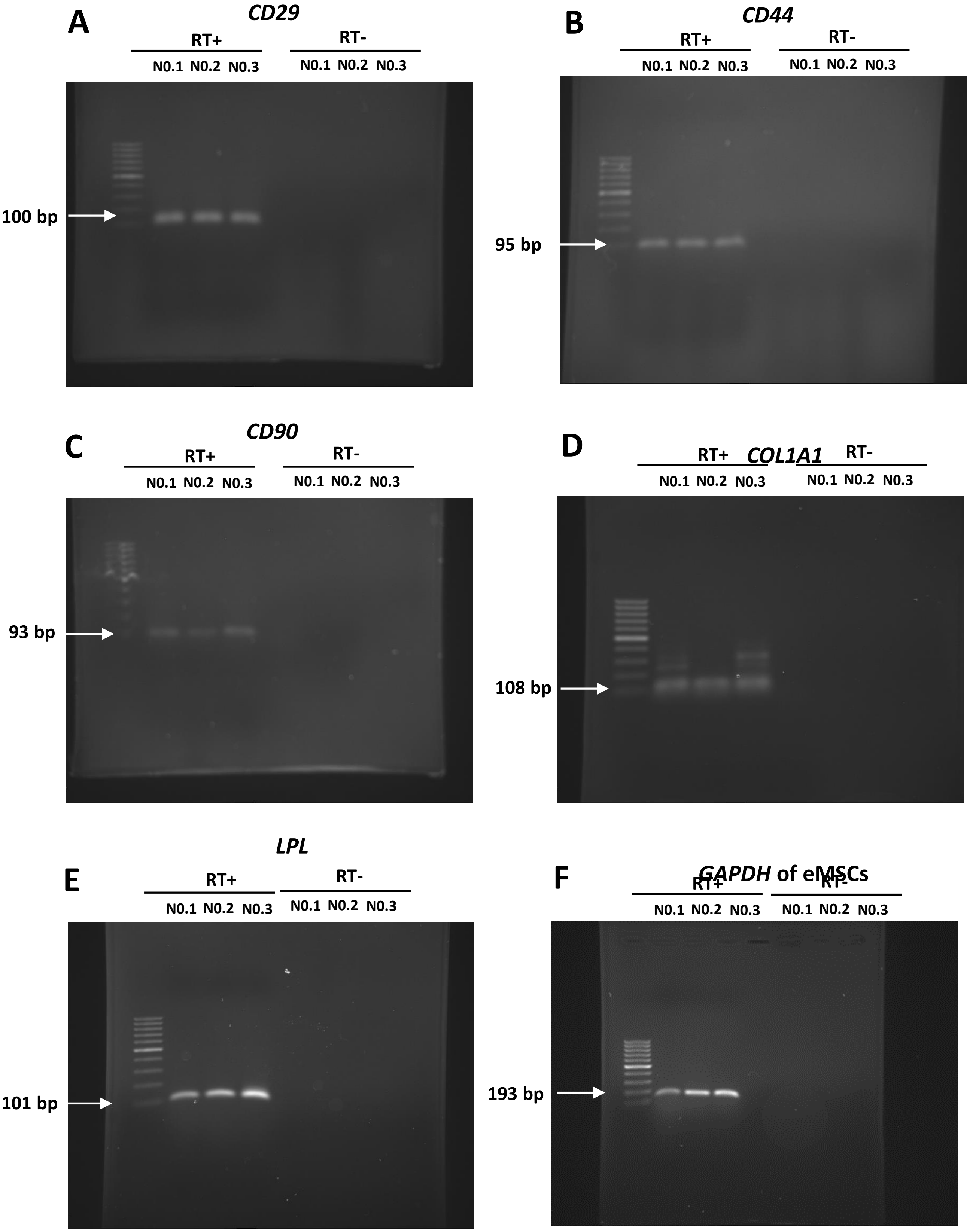


**Supplementary uncropped Figure 1**: The gel electrophoresis figures provided the original uncropped of gene-specific expression of the eMSCs in this study. (**A**), (**B**), and (**C**) exhibit gene-specific markers characteristic of eMSCs (*CD29*, *CD44*, and *CD90* respectively), while (**D**) depicted a marker specific to osteogenicity, and (**E**) highlights one specific to adipogenicity. Additionally, (**F**) showcases the expression pattern of a housekeeping gene in eMSCs.

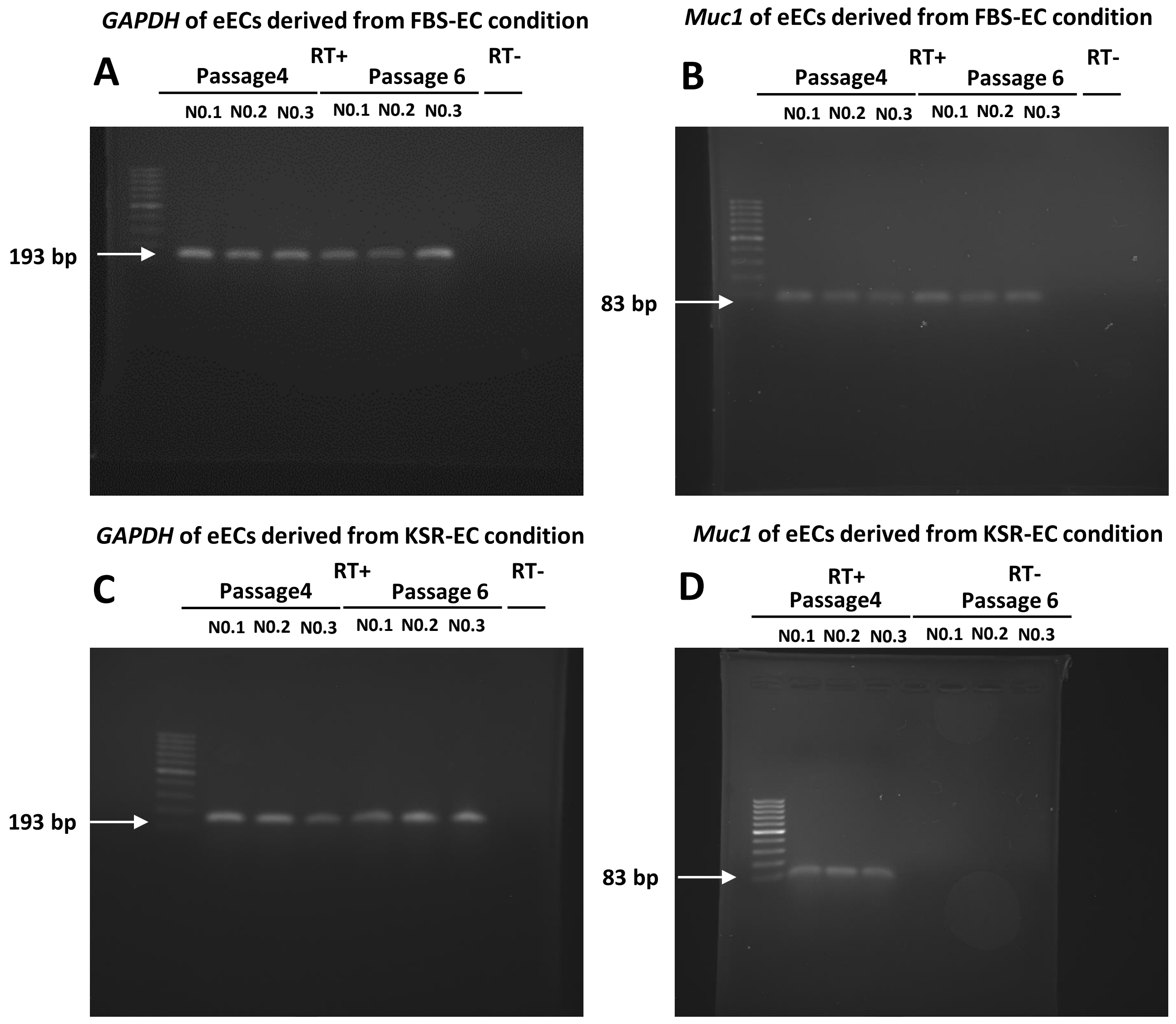


**Supplementary uncropped Figure 2**: (**A**, **B**) the uncropped gel electrophoresis of the housekeeping gene (*GAPDH*) and *Muc1* from eECs 3 cell lines under FBS-EC condition. (**C**, **D**) the uncropped gel electrophoresis bands of *GAPDH* and *Muc1* from the same 3 eECs cell lines under the KSR-EC condition are presented.

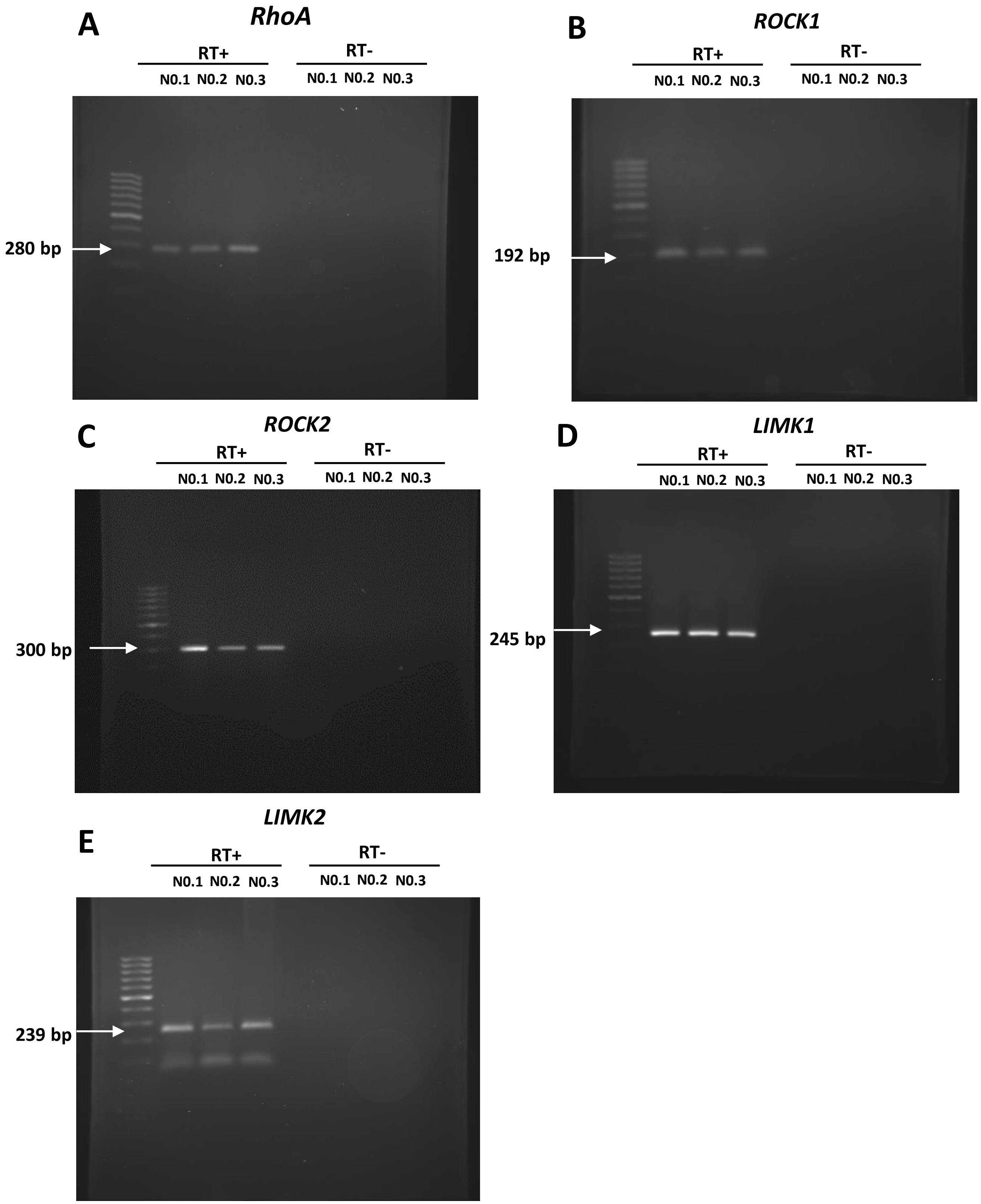


**Supplementary uncropped Figure 3:** the uncropped gel electrophoresis of the gene-specific Rho-associated protein kinase Pathway which was expressed in the eECs cell line. The genes were included (**A**) *RhoA*, (**B**) *ROCK1*, (**C**) *ROCK2*, (**D**) *LIMK1*, (**E**) *LIMK2*

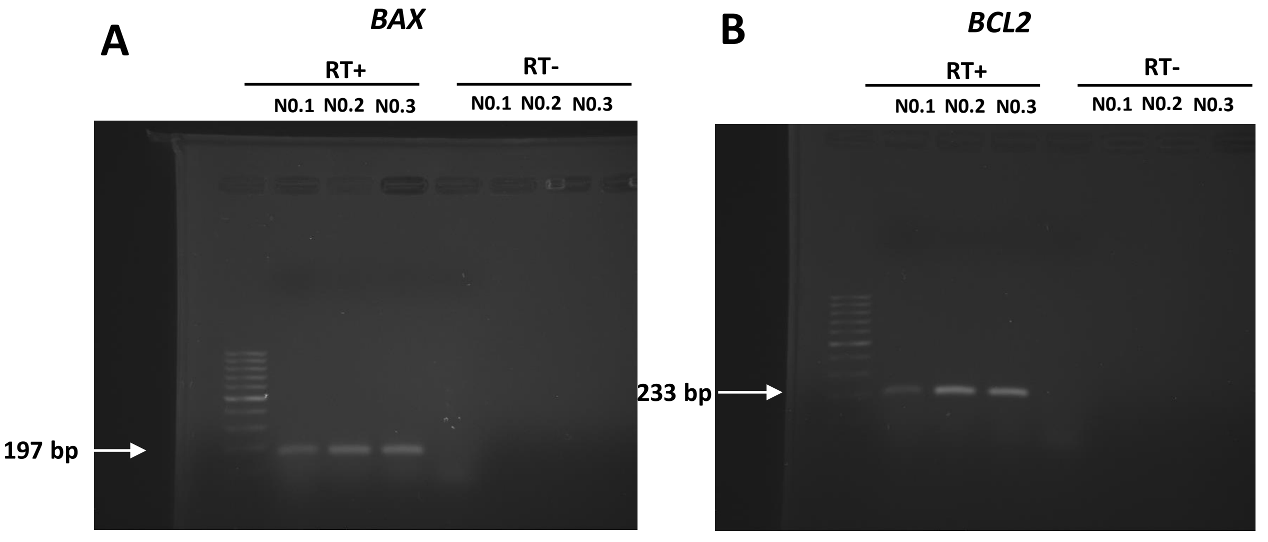


**Supplementary uncropped Figure 4:** The uncropped gel electrophoresis demonstrated the expression of genes related to the apoptosis pathway, specifically *BAX* (**A**) and *BCL2* (**B**), following apoptosis induced by $H_2O_2$ in the eECs cell line.

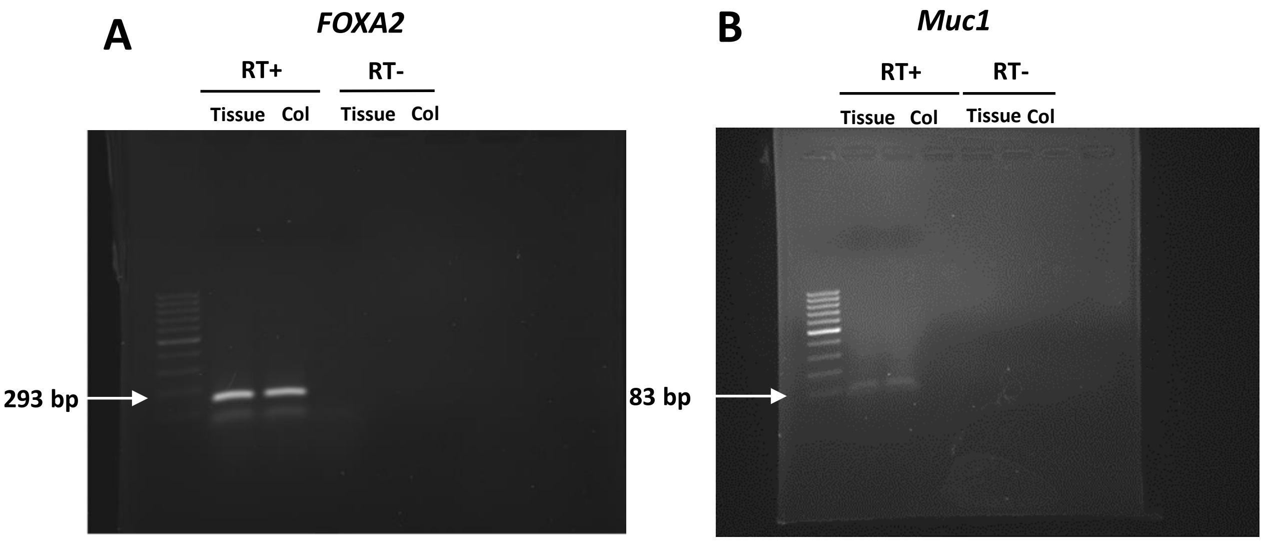


**Supplementary uncropped Figure 5:** The uncropped gel electrophoresis reveals the expression of the *FOXA2* gene (**A**) in both *in vivo* endometrial tissue and *in vitro* 3D-ET. Meanwhile, (**B**) demonstrated the *Muc1* gene expression in *in vitro* 3D-ET and *in vivo* tissue.
